# Specific Alterations in Astrocyte Properties via the GluA2-GAPDH Complex Associated with Multiple Sclerosis

**DOI:** 10.1038/s41598-018-31318-4

**Published:** 2018-08-27

**Authors:** Frankie H. F. Lee, Hailong Zhang, Anlong Jiang, Clement C. Zai, Fang Liu

**Affiliations:** 1 Campbell Family Mental Health Research Institute, Centre for Addiction and Mental Health, Toronto, Ontario M5T 1R8 Canada; 2 Department of Psychiatry, University of Toronto, Toronto, Ontario M5T 1R8 Canada; 3 Institute of Medical Science, University of Toronto, Toronto, Ontario M5T 1R8 Canada; 4 Laboratory Medicine and Pathobiology, University of Toronto, Toronto, Ontario M5T 1R8 Canada; 5 Physiology, University of Toronto, Toronto, Ontario M5T 1R8 Canada

## Abstract

There is strong evidence indicating neuroinflammation is an important mediator in multiple sclerosis (MS), with astrogliosis playing a significant role in this process. Surprisingly, astrocytes exert paradoxical roles during disease development, but the mechanisms remain unknown. Previously, we have reported that administering an interfering peptide (GluA2-G-Gpep) which specifically disrupts the GluA2-GAPDH interaction rescued neurological symptoms in the EAE mouse model of MS. In this study, we validated that the GluA2-GAPDH complex was elevated in LPS-induced primary reactive astrocytes, and GluA2-G-Gpep treatment significantly reduced GFAP expression levels in both EAE mice and reactive astrocytes. Further *in vivo* and *in vitro* analyses revealed that GluA2-G-Gpep administration normalized EAAT1 and EAAT2 expression, rescued compromised blood-brain barrier integrity via AQP4, promoted actin reorganization and changed mitochondrial dynamics. These alterations may partially be explained by changes in the nuclear GAPDH and p53 transcription pathways. Our findings provide critical implications for understanding the astrocyte properties regulated by GluA2-GAPDH associated with MS, and insights for novel treatment options targeting at astrocytes.

## Introduction

Multiple sclerosis (MS) is a neuroinflammatory disorder of the central nervous system (CNS) with accumulating evidence implicating that CNS inflammation is a primary determinant of damage in MS^1,2^. However, its unknown etiology along with the complexity of this disease produces a significant challenge in delineating the exact pathological mechanisms. In recent years, researchers have suggested that microglia, peripheral immune cells and astrocytes play critical roles in MS disease progression. In particular, initial pathological studies have reported that astrocytes morphology was highly abnormal in early active MS lesions^3^. Astrocytes are the most abundant cell types in the mammalian CNS, where they contribute to multiple physiological functions, including the regulation of extracellular ions and neurotransmitters concentrations, synthesis of neuronal metabolic substrates, removal of neurotoxic substances, and formation and pruning of synapses^4,5^.

Astrogliosis is a ubiquitous feature of CNS pathologies, which occurs in response to neurological insults. Reactive astrocytes show distinctive morphological and functional changes including upregulation of glial fibrillary acidic protein (GFAP), hypertrophy and proliferation, progressive alterations in gene and protein expression, and scar formation^6–8^. In the context of MS, astrocytes could contribute by playing an active role as part of the immune system with induction of inflammatory cytokines, modulation of blood-brain barrier (BBB) integrity, uptake of excess glutamate, release of factors affecting axon myelination, regeneration and viability of neurons and oligodendrocytes, and altering mitochondrial functions^3^. Surprisingly, astrocytes in MS lesions exert paradoxical roles during disease development^9,10^. For example, there is evidence demonstrating that astrocytes secrete pro-inflammatory mediators leading to inhibition of axon remyelination and regeneration, and disruption of BBB, thus allowing the recruitment of T cells, macrophages and microglia to lesion sites^3,11,12^. In contrast, other studies have reported protective roles of astrocytes in secreting anti-inflammatory cytokines, factors that promote neuron and oligodendrocyte survival, proliferation and differentiation, and prevention of excitotoxicity by glutamate uptake^3,11,13,14^. Better knowledge of this dichotomy observed in astrocytic effects and their specific roles in MS pathophysiology would provide important insights in designing new therapeutic strategies targeting astrocytes.

AMPA-type receptors (AMPARs) are the primary mediators of fast excitatory synaptic transmission in the mammalian central nervous system and are expressed on both neurons and astrocytes^15^. The GluA2 subunits of the AMPARs, also expressed in astrocytes, are crucial determinants in controlling the biophysical properties of calcium permeability, receptor kinetics and channel conductance^15–17^. In our previous studies, we have identified a novel interaction between GluA2 and glyceraldehyde 3-phosphate dehydrogenase (GAPDH), where this complex plays vital roles in various cell functions, such as cell death and neuronal growth^18–20^. Interestingly, abnormally enhanced levels of the GluA2-GAPDH complex were found in both human MS plaques and experimental autoimmune encephalomyelitis (EAE) mice, an animal model of MS^21^. Treatment with GluA2-G-Gpep, an interfering peptide that specifically disrupts this interaction, resulted in substantial rescue of neurological symptoms in EAE mice^21^. These effects are mediated by the internalization of GluA2 and GAPDH instead of affecting AMPA receptor functions. It is likely that the GluA2-GAPDH interaction may also have significant impact towards astrocyte functions as supported by the described evidence. Therefore, we investigated the specific changes in astrocyte morphology, protein expressions and functions regulated by this interaction. These findings will help us better understand the role of GluA2-GADPH in astrocytes associated with MS, and provide crucial information in determining the astrocytic changes that could be beneficial to MS treatment.

## Results

### Disrupting GluA2-GAPDH interaction with GluA2-G-Gpep reduces astrocytes reactivity in the EAE mice

Many studies have provided strong evidence for the involvement of reactive astrocytes in the pathological mechanisms of MS^3,11^, but how astrogliosis is regulated and contribute to disease symptoms remains unknown. In this study, we examined the potential roles of the GluA2-GAPDH complex in modulating astrogliosis using the EAE mouse model of MS. The presence of GluA2 subunits and its colocalization with GAPDH in GFAP-positive astrocytes was identified and confirmed by immunofluorescence on mouse spinal cords and primary astrocytes, as well as co-immunoprecipitation using astrocytic proteins (Supplementary Fig. 1a,b). TAT-GluA2-G-Gpep is an interfering peptide that was developed encoding the binding region of GluA2 in interacting with GAPDH, hence it can specifically disrupt GluA2-GAPDH complex formation by competing with GluA2 for GAPDH^19^. Furthermore, recombinant proteins fused to the cell membrane transduction domain TAT have been used extensively for efficient delivery of full-length functional proteins into animals *in vivo*, with great success in crossing the blood-brain barrier and cell membrane^22–24^. Besides, cytotoxicity assays revealed negligible effects of TAT up to concentrations of 50 µM^25^.

**Figure 1:**
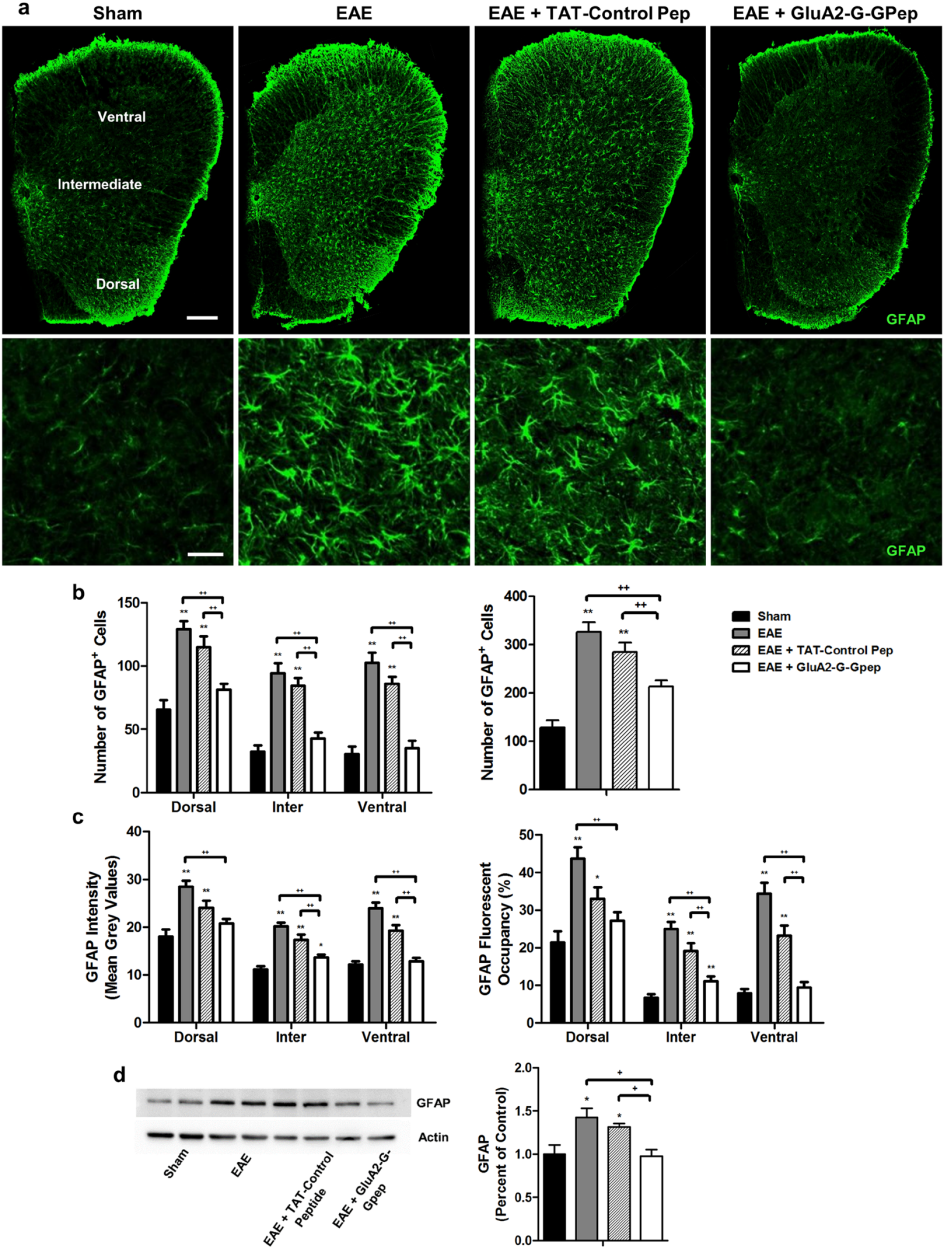
Disrupting GluA2-GAPDH interaction with GluA2-G-Gpep reduces astrocytes reactivity in the EAE mice. (**a**) Representative fluorescent images showing GFAP-labeled astrocytes in spinal cord sections of sham, EAE, EAE with TAT-control peptide (10 μM) and EAE with GluA2-G-Gpep (10 μM) mice. Scale Bar: 100 μm. Higher magnification images are shown below. Scale Bar: 20 μm. The analyzed regions include dorsal, intermediate and ventral grey matter. **(b**) There was a significant increase in the number of GFAP^+^ cells in the EAE and EAE with TAT-control peptide groups when compared sham controls in all spinal cord regions. GluA2-G-Gpep treatment produced a marked reduction in GFAP-labeled cell numbers comparable to controls. (**c**) Astrocyte reactivity was quantified using mean grey values of fluorescent intensity, and measured as percent area occupancy from a normalized thresholding scale with ImageJ. EAE and EAE + TAT-control pep mice had significantly higher fluorescence intensity values and fluorescent occupancy vs. sham animals, while administration of GluA2-G-Gpep reduced both parameters (Sham: n = 12; EAE: n = 10; TAT-Control Pep: n = 16; GluA2-G-Gpep: n = 13 sections from 3 different spinal cords, two-way ANOVA followed by Bonferroni *post hoc* test). **(d)** The changes in GFAP expression among the different groups were confirmed with Western blot experiments (n = 4 spinal cords per group, one-way ANOVA followed by Bonferroni *post hoc* test). The full-length blot is shown and quantification of protein expression was normalized with actin loading controls and expressed as a percentage of sham groups. Data are presented as mean ± SEM. *p < 0.05, **p < 0.01 vs. sham, ^+^*p* < 0.05, ^++^*p* < 0.01 vs. EAE with GluA2-G-Gpep.

Immunohistochemistry was performed on lumbar spinal cord sections of EAE mice using an antibody against GFAP, which is a standard marker for labeling astrocytes. Representative fluorescent images of spinal cord sections from different groups are shown in Fig. 1a with higher magnification images displayed below. As expected, we observed significantly more GFAP-positive astrocytes in EAE mice when compared to sham for all the different spinal cord regions (Fig. 1a,b). The degree of astrocyte reactivity is positively correlated with GFAP expression^7,26^, hence measuring GFAP fluorescent intensities would indicate the state of astrogliosis. Indeed, a similar pattern of higher GFAP intensity and fluorescent occupancy was detected in the EAE group (Fig. 1c). Remarkably, GluA2-G-Gpep treatment significantly reduced GFAP^+^ cell numbers and GFAP expression levels within each spinal cord region of EAE mice, while TAT-control peptide had no effect (Fig. 1a–c). Western blot analysis from mouse spinal cord tissues of different treatment groups further verified the changes in GFAP protein expression, consistent with the immunofluorescence data (Fig. 1d). These results indicate that disrupting the GluA2-GAPDH interaction with GluA2-G-Gpep in the EAE mouse could normalize astrocyte reactivity.

### GluA2-G-Gpep treatment alters the expression pattern of astrocyte-specific AQP4 and rescues compromised blood-brain barrier permeability displayed in the EAE mouse

To first demonstrate the involvement of astrocytes in MS, we analyzed the expression of other proteins that have been reported to be expressed in astrocytes and/or associated with astrocytic changes in the EAE mouse. Western blot results showed that vimentin, GAP43, 14-3-3ε, NCAM1, AQP4 and EAAT1 levels were all significantly increased while α-tubulin was decreased in EAE mouse spinal cords (Supplementary Fig. 2). Moreover, based on two independent genome-wide association studies (GWAS), there was a consistent association of single-nucleotide polymorphisms in the identified genes with MS (Supplementary Table 1). These findings provide the basis to support that alterations in astrocytic functions are crucial in the MS setting.

**Figure 2:**
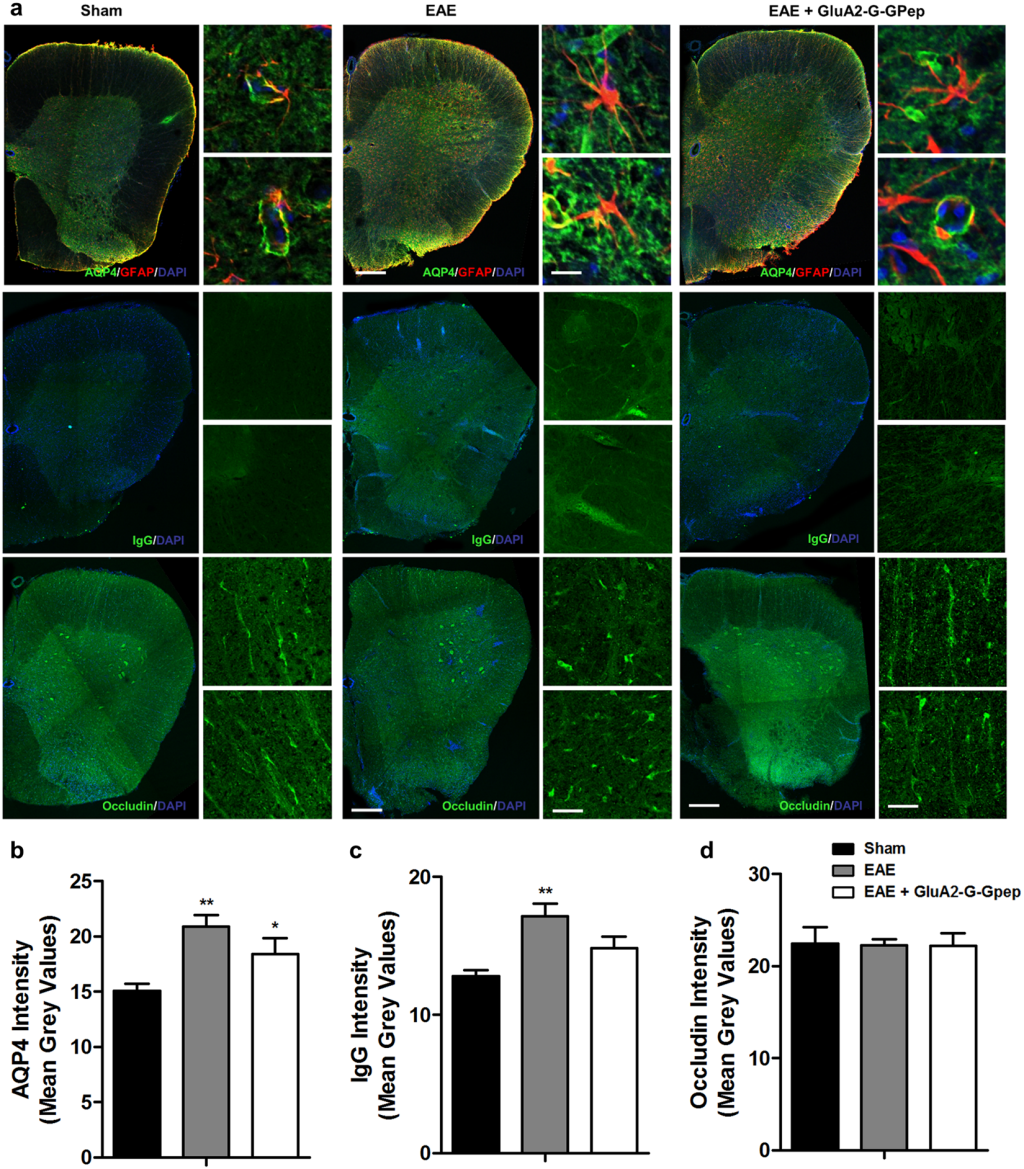
GluA2-G-Gpep treatment alters the expression pattern of astrocyte-specific AQP4 and rescues compromised blood-brain barrier permeability displayed in the EAE mouse. (**a**) Immunohistochemistry of spinal cord sections labeling AQP4/GFAP, IgG and occludin in sham, EAE and EAE with GluA2-G-Gpep mice. Higher magnification images shown in the right. Scale Bar: 100 μm (left), 20 μm (right). AQP4 was mainly expressed in a circular pattern of GFAP-astrocytes in sham mice, but EAE groups had a more widespread distribution that was not confined to astrocytes. Blood-brain barrier integrity was analyzed by IgG extravasation and occludin expression pattern in the mouse spinal cord. EAE mice exhibited prominent IgG expression and disrupted occludin tight junction structure vs. sham controls. Surprisingly, these changes were reversed with GluA2-G-Gpep treatment. (**b**) Quantification of the fluorescence signal intensities showed a significant increase with AQP4 and IgG in EAE mice, but GluA2-G-Gpep produced only a slight reduction. Occludin expression levels were not different among the groups. (Sham: n = 8; EAE: n = 6–8; GluA2-G-Gpep: n = 7–8 sections from 3 different spinal cords, one-way ANOVA followed by Bonferroni *post hoc* test). Data are presented as mean ± SEM. *p < 0.05, **p < 0.01 vs. sham.

Aquaporin 4 (AQP4) is a water-channel protein that is primarily expressed on astrocyte end feet in contact with capillaries, and is critical in the composition of the BBB^27,28^. Therefore, astrocytes are strongly associated with the regulation of BBB functions and maintenance of its integrity. An important event in MS and neuroinflammation is the diminished barrier function of the BBB which facilitates other inflammatory mediators to be effectively transported into the damaged regions^29^. We investigated whether this property is disturbed in the EAE mice and the effects of GluA2-G-Gpep administration. Immunohistochemistry with AQP4 revealed a higher fluorescence expression in the EAE mice when compared to sham (Fig. 2a,b). AQP4 localization was mainly in a circular pattern of GFAP-astrocytes in sham mice, while EAE groups had a more widespread distribution that was not confined to astrocytes (Fig. 2a). AQP4 fluorescence intensity did not change after GluA2-G-Gpep treatment in the EAE mice, but showed a similar expression pattern as observed with sham groups (Fig. 2a,b). To further evaluate BBB permeability alterations, we conducted IgG extravasation and immunolabelling of occludin, which is a tight junction protein involved in BBB. As expected, we found negligible amount of IgG present in sham groups in contrast to the prominent expression observed in EAE mice. GluA2-G-Gpep administration produced a marginal decrease in IgG levels (Fig. 2a,c). Finally, occludin immunofluorescence intensity remained similar among the different groups. However, EAE spinal cords displayed disorganized tight junction structures, possibly indicative of BBB disruption. Occludin in peptide-treated mice showed continuous expression in a longitudinal pattern, analogous to sham controls (Fig. 2a,d). These data provide evidence for the role of GluA2-GAPDH complex in modulating AQP4 expression and BBB functions.

### GluA2-G-Gpep lowers the increase in EAAT1 and EAAT2 expression within EAE mice

**Figure 3:**
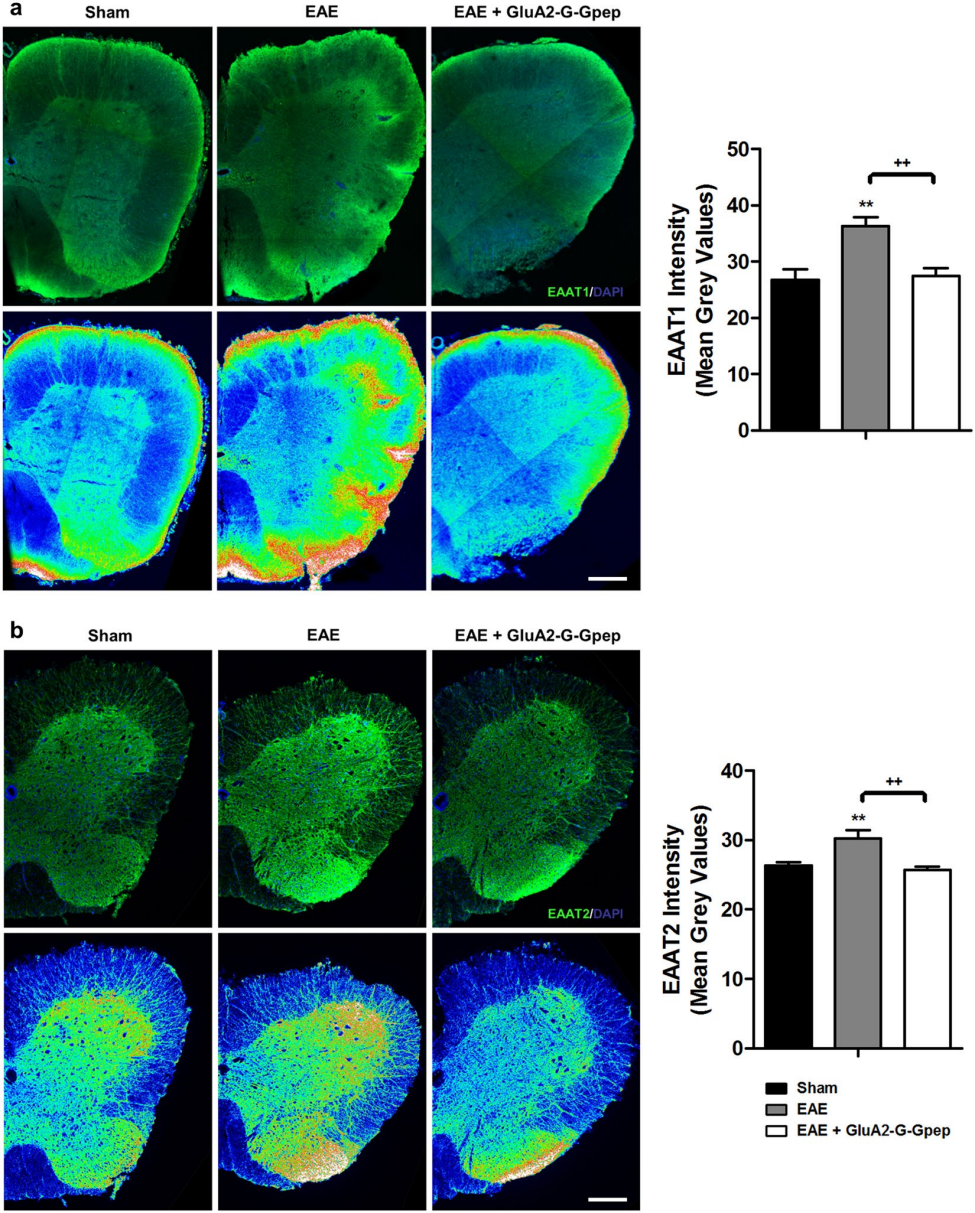
GluA2-G-Gpep treatment effectively reduces the increase in EAAT1 and EAAT2 expression levels within EAE mice. **(a,b**) Immunofluorescent images of EAAT1 and EAAT2 of sham, EAE and EAE with GluA2-G-Gpep mouse spinal cords. Heat maps of fluorescence intensities are shown below. Scale Bar: 100 μm. There was a marked increase in both EAAT1 and EAAT2 fluorescence signals with the EAE groups when compared to sham, but administration of GluA2-G-Gpep significantly reduced the transporters levels comparable to controls. Quantification of EAAT1 and EAAT2 fluorescence intensities resulted in a similar trend for all groups. (Sham: n = 10–12; EAE: n = 10; GluA2-G-Gpep: n = 12 sections from 3 different spinal cords, one-way ANOVA followed by Bonferroni *post hoc* test). Data are presented as mean ± SEM. **p < 0.01 vs. sham, ^++^*p* < 0.01 vs. EAE with GluA2-G-Gpep.

The astrocytic glutamate transporters EAAT1 (GLAST) and EAAT2 (GLT1) are also primarily expressed in astrocytes, where they are essential in maintaining normal excitatory neurotransmission and preventing excitotoxicity by removing excess glutamate during neuroinflammation^30^. Earlier studies have demonstrated an increased expression and function of EAAT1 and EAAT2 in MS as a regulatory response to toxic levels of glutamate in the CNS^31^. Here, we examine the effects of GluA2-GAPDH complex on these astrocyte glutamate transporters in the EAE mouse. EAAT1 and EAAT2 immunostaining on spinal cord sections resulted in greater fluorescence signal intensities in EAE groups vs. controls as shown with heat maps of EAAT1 and EAAT2 fluorescence (Fig. 3a,b). Surprisingly, the addition of GluA2-G-Gpep normalized the increased expression of both transporters back to control levels (Fig. 3a,b), suggesting that disruption of GluA2-GAPDH interaction could affect astrocyte-specific glutamate transporters in MS.

### Disrupting GluA2-GAPDH interaction directly affects astrocytes morphology and reactivity in primary astrocyte cultures

It is important to note that our *in vivo* analyses of protein expression involve the use of whole spinal cord sections and tissues, which may not be selective for astrocytes. Moreover, it remains unclear whether the reduction of reactive astrocytic response and change in astrocytic functions are direct effects of blocking GluA2-GAPDH interaction on astrocytes or an indirect outcome of other compensatory pathways. To address this issue, we mimicked astrogliosis condition by challenging primary astrocytes with lipopolysaccharide (LPS), which is well-documented in stimulating astrocyte activity^8^. The enhanced GluA2-GAPDH interaction in LPS-stimulated astrocytes was validated, and GluA2-G-Gpep treatment effectively disrupted this complex formation but not with TAT-control peptide (Supplementary Fig. 1b).

**Figure 4:**
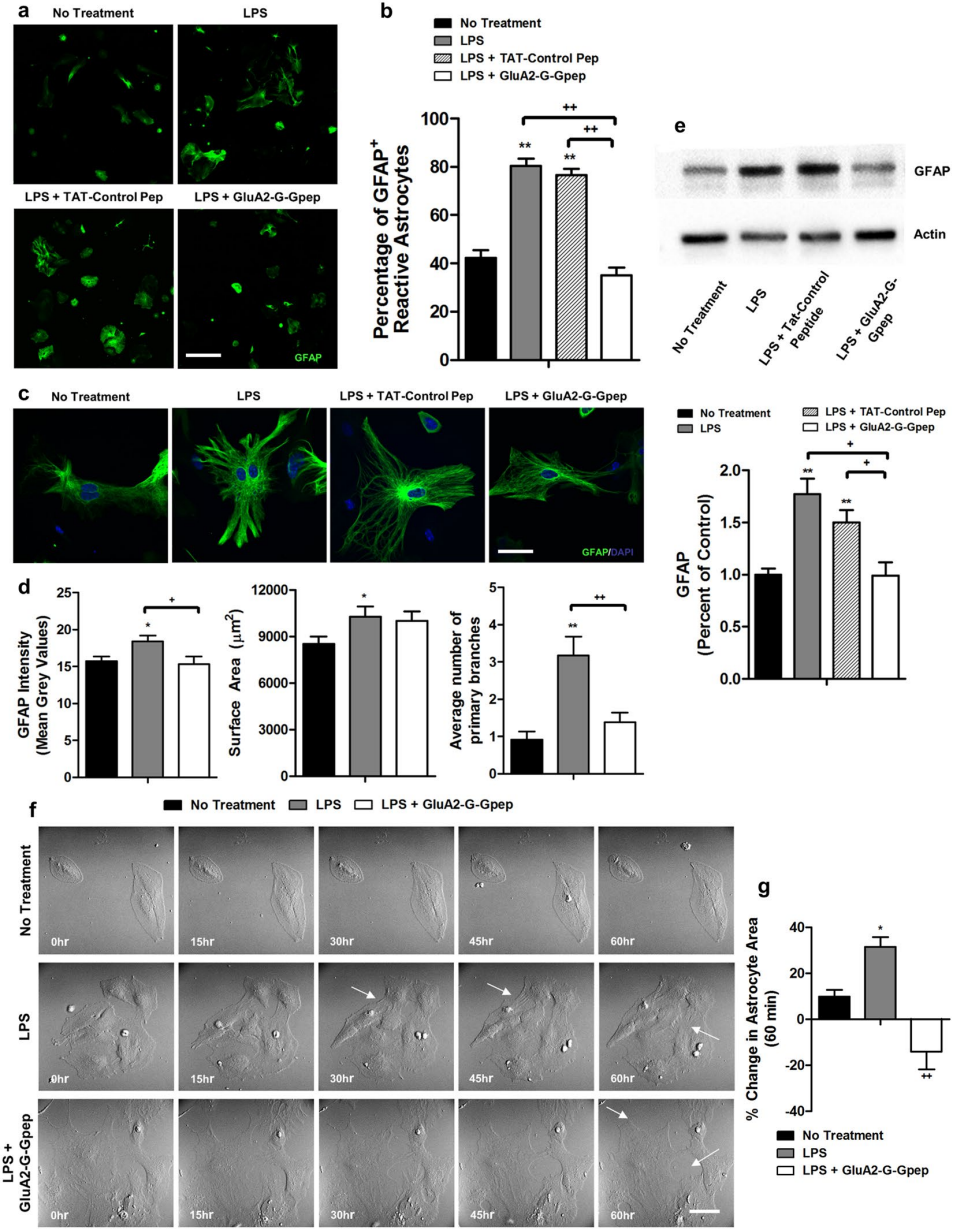
Disrupting GluA2-GAPDH interaction directly affects astrocytes morphology and reactivity in primary astrocyte cultures. **(a**) Immunostaining of GFAP was performed on primary astrocyte cultures and fluorescent images were captured at a low magnification 10x to provide an overall view of astrocyte cultures. Scale Bar: 100 μm. **(b)** The percentage of GFAP^+^ cells was measured as the number of GFAP-labeled cells per total number. There was a significantly higher proportion of reactive astrocytes with LPS and LPS with TAT-control peptide when compared to no treatment controls, but GluA2-G-Gpep treatment effectively reduced this increase (No Treatment: n = 15; LPS: n = 13; TAT-Control Pep: n = 13; GluA2-G-Gpep: n = 12 ROIs from 3 different cultures, one-way ANOVA followed by Bonferroni *post hoc* test). **(c)** Higher magnification images at 60 × of individual astrocytes immunostained with GFAP were captured for morphological analyses. Scale Bar: 20 μm. **(d**) There was a pronounced increase in GFAP fluorescent intensity, astrocyte surface area and the number of primary branches in LPS-stimulated astrocytes vs. control group. GluA2-G-Gpep administration decreased GFAP intensity and the number of primary processes, but not astrocyte surface area (No Treatment: n = 22; LPS: n = 23; GluA2-G-Gpep: n = 25 astrocytes from 3 different cultures, one-way ANOVA followed by Bonferroni *post hoc* test). **(e**) Western blot analysis confirmed that GFAP protein expression was higher in LPS-induced astrocytes than non-treated cells, and GluA2-G-Gpep treatment was able to revert its expression back to control levels (n = 4 different cultures per group, one-way ANOVA followed by Bonferroni *post hoc* test). Full-length Western blots are presented in Supplementary Fig. 4a. **(f)** Time-lapse imaging of primary astrocytes further revealed more processes with strong development of intermediate filaments in LPS-induced astrocytes. Consistently, GluA2-G-Gpep treatment normalized this phenotype with less filaments developed. White arrows indicate the change in astrocyte processes. Scale Bar: 20 μm. **(g)** Quantification of the percent change in astrocyte surface area between time 0 and 60 hours showed a significant increase with LPS treatment, but was decreased upon GluA2-G-Gpep addition (No Treatment: n = 7; LPS: n = 7; GluA2-G-Gpep: n = 6 cells from 3 different cultures, one-way ANOVA followed by Bonferroni *post hoc* test). Data are presented as mean ± SEM. *p < 0.05, **p < 0.01 vs. no treatment, ^+^*p* < 0.05, ^++^*p* < 0.01 vs. LPS with GluA2-G-Gpep.

Astrogliosis is characterized by profound molecular and morphological changes in astrocytes in response to all CNS injuries and diseases. For example, a marked increase in GFAP and vimentin expression is commonly seen in reactive astrocytes. Morphologically, astrocytes undergo extensive hypertrophy and proliferation, together with more complex branching patterns^4,7^. Therefore, we examined whether GluA2-GAPDH disruption would have a direct effect on reversing astrocytes reactivity and morphology using primary astrocyte cultures. There was a significantly higher proportion of GFAP^+^ reactive astrocytes with LPS treatment when compared to no treatment and TAT-control peptide groups, but the numbers were reduced back to control level under GluA2-G-Gpep treatment (No treatment: 42.28 ± 3.23%; LPS: 80.25 ± 3.09%; LPS + TAT-Control: 76.56 ± 2.56%; LPS + GluA2-G-Gpep: 34.97 ± 3.33%) (Fig. 4a,b). At the individual cell level, LPS-induced astrocytes displayed a profound increase in GFAP intensity, astrocyte surface area and average number of astrocytic primary branches than controls. GluA2-G-Gpep administration markedly normalized GFAP intensity and the number of primary processes comparable to control groups, but had no effect on astrocyte surface area (GFAP Intensity - No treatment: 15.69 ± 0.66; LPS: 18.39 ± 0.79; GluA2-G-Gpep: 15.31 ± 1.04; Surface area - No treatment: 8534.08 ± 474.54 μm^2^; LPS: 10280.27 ± 675.41 μm^2^; GluA2-G-Gpep: 10002.25 ± 630.17 μm^2^; Primary branches - No treatment: 0.91 ± 0.23; LPS: 3.17 ± 0.51; GluA2-G-Gpep: 1.38 ± 0.26) (Fig. 4c,d). Western blot analysis confirmed the increased GFAP expression in astrocyte cultures with LPS treatment and GluA2-G-Gpep treatment significantly reduced GFAP levels (Fig. 4e). These data indicate that blocking the enhanced GluA2-GAPDH interaction can reverse reactive astrocytes back to resting state. To directly demonstrate that GluA2-GAPDH disruption can affect astrocyte morphology, time-lapse imaging was used to capture the growth pattern of primary astrocytes under different treatments (Fig. 4f). We measured the percent change of astrocyte area between 0 and 60 hours, and found a significant increase of 31.34% in LPS-induced astrocytes compared to 9.75% in control cells (Fig. 4g). Consistently, astrocytes with GluA2-G-Gpep addition after LPS induction showed a reduction of 14.12% of area (Fig. 4g). Moreover, there were significantly more processes in the LPS group, but cells exhibited less branches after treating with GluA2-G-Gpep, providing further evidence for the regulation of astrocyte reactivity by the GluA2-GAPDH complex.

Actin reorganization is a main determinant of cellular morphology, and has been shown to be responsible for the morphological changes in reactive astrocytes. There is evidence illustrating the loss and disassembly of F-actin fibres present in different models of astrogliosis^32,33^. We determined whether blocking GluA2-GAPDH interaction would reorganize actin fibres in astrocytes back to normal patterns. As shown in Supplementary Fig. 3, F-actin in non-treated astrocytes displayed a well-organized ring shape near the outer edge of the cell. In the presence of LPS stimulation, F-actin formed clusters in the middle part of the cell, but GluA2-G-Gpep treatment reversed this alteration of actin cytoskeleton, similar to quiescent astrocytes (Supplementary Fig. 3). These results illustrate that changes in astrocyte morphology observed with GluA2-GAPDH disruption may act via reorganizing F-actin structure mechanisms.

### Enhanced EAAT1 and EAAT2 expression is normalized with GluA2-G-Gpep in primary reactive astrocytes, but not AQP4

**Figure 5:**
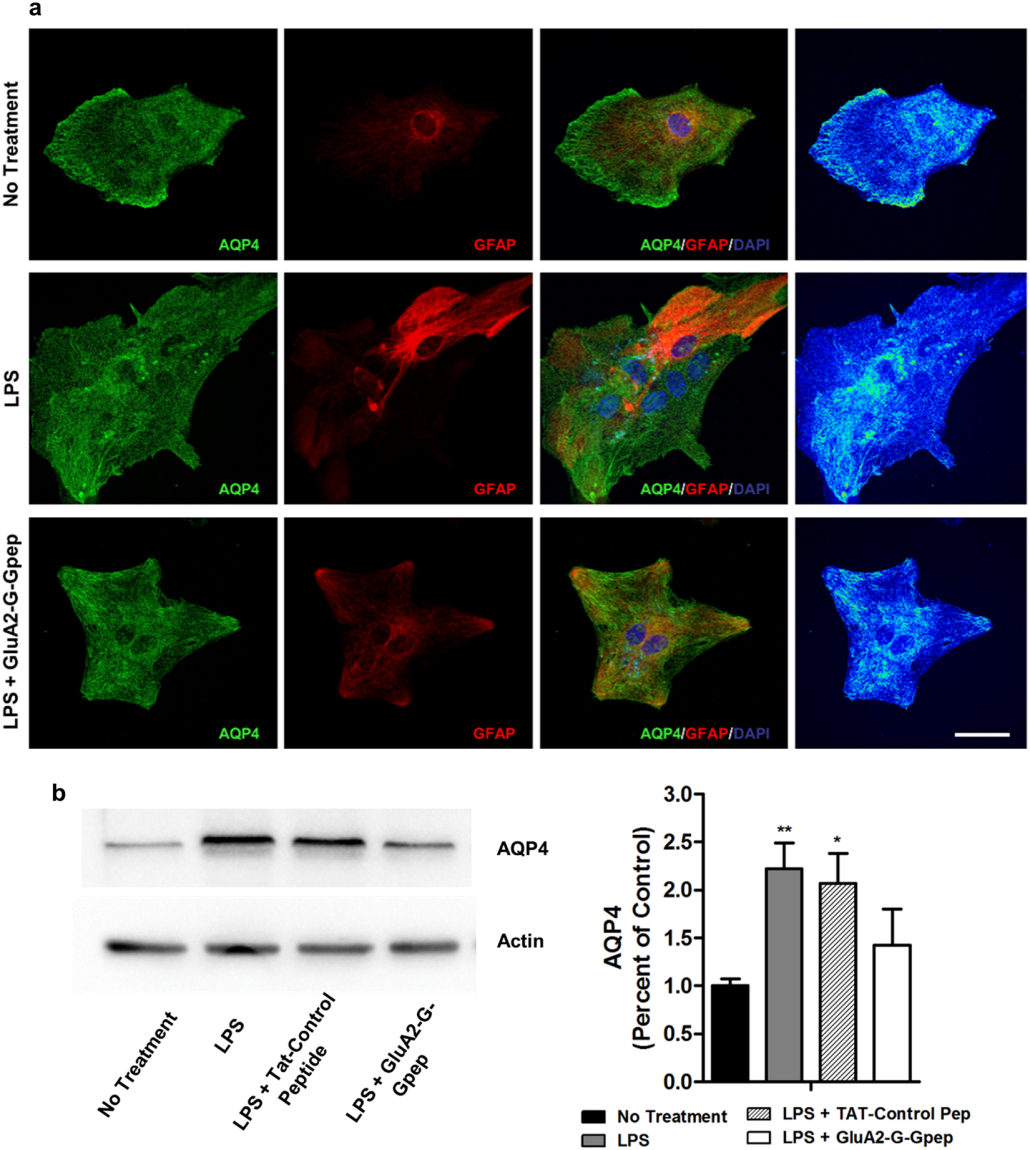
GluA2-G-Gpep treatment partially alters AQP4 expression pattern in primary astrocytes, but did not affect AQP4 levels. (**a)** Representative fluorescent images of immunocytochemistry using antibodies against AQP4 on astrocytes with LPS and LPS + GluA2-G-Gpep treatments. Heat intensity map of AQP4 fluorescence revealed AQP4 localized near the edge of the cell and within the cytoplasm in non-treated cells. After LPS stimulation, there were more AQP4 shifted towards cytoplasmic and nuclear regions. Treatment of GluA2-G-Gpep resulted in a similar distribution of AQP4 near the nucleus and within the cytoplasm, but less in the perimeter regions. Scale Bar: 20 μm. **(b)** Western blot results indicate that AQP4 protein levels were significantly increased in LPS-stimulated astrocytes when compared to non-treatment controls. GluA2-G-Gpep addition did not reverse this enhancement back to control levels despite exhibiting a decreasing trend (n = 4 different cultures per group, one-way ANOVA followed by Bonferroni *post hoc* test). Full-length Western blots are presented in Supplementary Fig. 4a. Data are presented as mean ± SEM. *p < 0.05, **p < 0.01 vs. no treatment.

Our next aim was to investigate the intracellular localization and expression of AQP4, EAAT1 and EAAT2 within primary astrocytes under LPS and GluA2-G-Gpep treatment. Immunostaining of AQP4 in primary astrocytes revealed that its intracellular localization is both near the edge of the cell and within the cytoplasm in non-treated condition. After LPS stimulation, there was more AQP4 shifted towards the cytoplasm and nuclear region of the cell (Fig. 5a). GluA2-G-Gpep-treated astrocytes showed a similar distribution of AQP4 near the nucleus and within the cytoplasm, but less in the perimeter regions (Fig. 5a). Total AQP4 expression was significantly increased with LPS stimulation, while GluA2-G-Gpep addition did not reverse this enhancement back to control levels despite exhibiting a decreasing trend (Fig. 5b).

**Figure 6:**
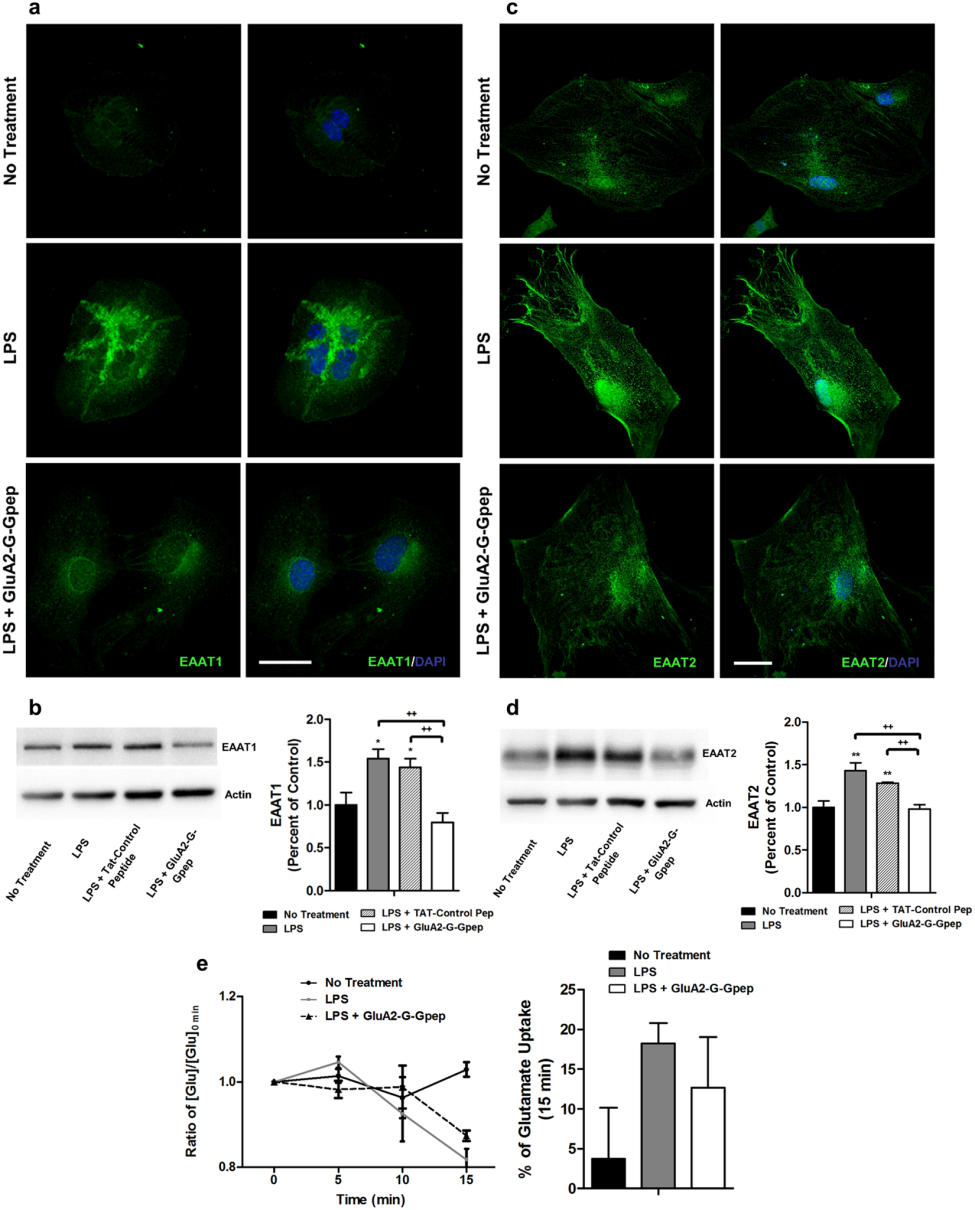
GluA2-G-Gpep reduces the enhanced EAAT1 and EAAT2 expression in LPS-induced reactive astrocytes, but did not affect glutamate uptake activity. (**a**,**c**) Immunofluorescent images of EAAT1 and EAAT2 in primary astrocytes revealed a pronounced increase in both transporters expression within the cell, but was markedly reduced after administration of GluA2-G-Gpep. Scale Bar: 20 μm. (**b**,**d**) Quantification of EAAT1 and EAAT2 protein levels by Western blot produced consistent results with LPS-reactive astrocytes showing higher expression and peptide-treated groups were rescued back to normal levels (n = 4 different cultures per group, one-way ANOVA followed by Bonferroni *post hoc* test). (**e**) Glutamate uptake function in primary astrocytes was assessed by measuring glutamate concentration at 0, 5, 10 and 15 min after addition of 50 μM of extracellular glutamate. There was a modest decrease in glutamate concentration for LPS and LPS + GluA2-G-Gpep group after 10 and 15 min. When calculating the percent reduction of glutamate at 15 min, we observed a higher percent with the LPS-astrocytes, but peptide treatment did not alter uptake activity (n = 3 different cultures per group, one-way ANOVA followed by Bonferroni *post hoc* test). Glutamate concentration experiments were performed in duplicates for each sample. All data among the different groups did not reach statistical significance. Full-length Western blots of (**b**,**d**) are presented in Supplementary Fig. 4a. Data are presented as mean ± SEM. *p < 0.05, **p < 0.01 vs. no treatment, ^++^*p* < 0.01 vs. LPS with GluA2-G-Gpep.

As for glutamate transporters, we observed a significant increase in both EAAT1 and EAAT2 expression in astrocytes after LPS challenge, and GluA2-G-Gpep administration effectively reduced their levels comparable to controls (Fig. 6a,c). Western blots with astrocyte proteins of different treatments also coincided with these changes (Fig. 6b,d). Furthermore, we measured the glutamate uptake ability in primary astrocytes and found that glutamate concentration was only slightly reduced by 18% after 15 min in LPS-treated cells (Fig. 6e). However, GluA2-G-Gpep treatment did not produce any significant change in glutamate concentration, indicating that glutamate removal was no different than the LPS group (Fig. 6e). Together, our *in vitro* findings support that GluA2-GAPDH disruption could alter AQP4 localization and EAAT1/2 expression.

### GluA2-GAPDH disruption reverts mitochondria morphology in reactive astrocytes

**Figure 7:**
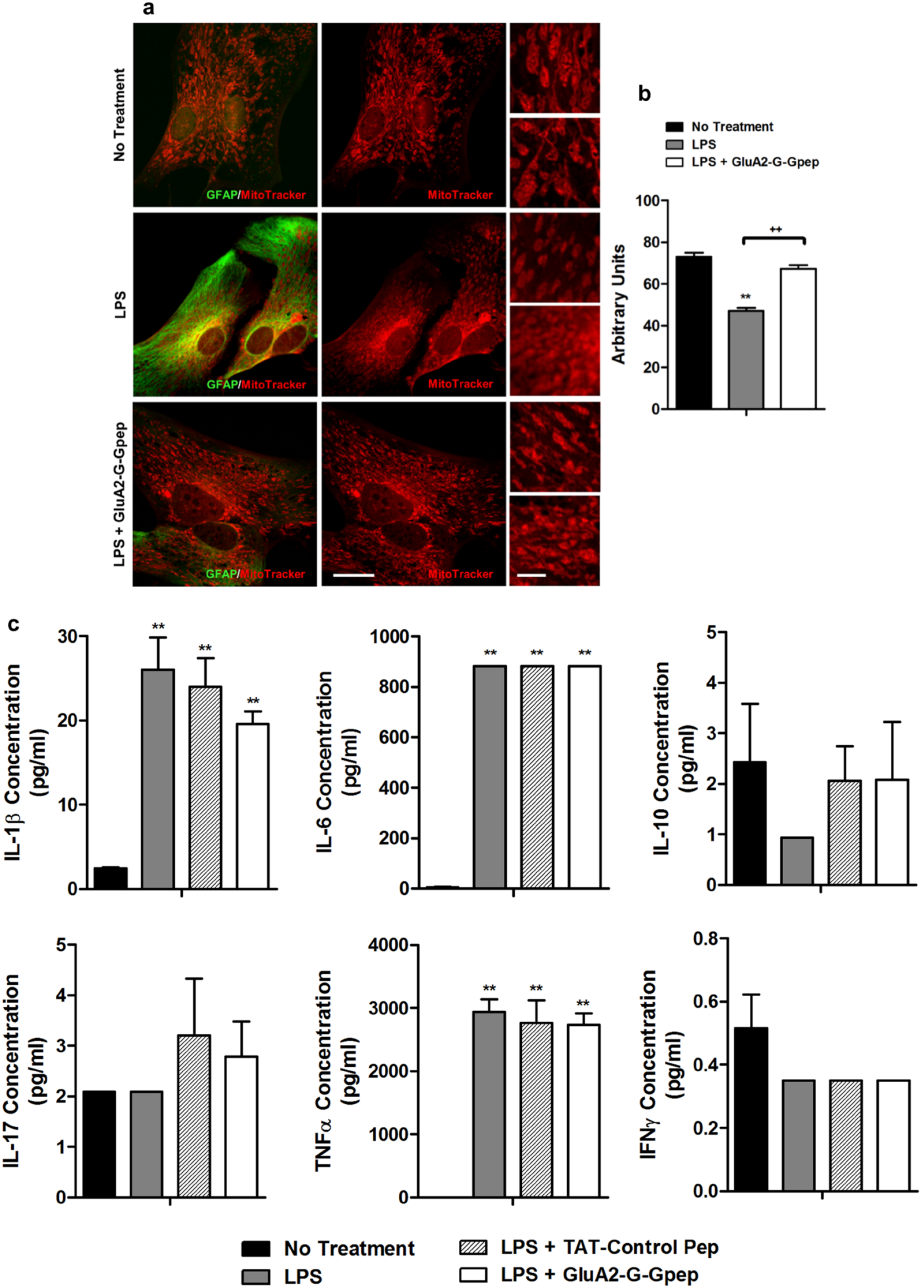
GluA2-GAPDH disruption reverts mitochondria morphology in reactive astrocytes but had no effects on cytokine release. (**a)** Representative fluorescent images showing mitochondrial morphology in primary astrocytes using mitotracker red. Higher magnification of mitochondria are shown on the right panels. LPS-treated astrocytes showed significantly smaller mitochondria, a feature of mitochondrial fission, when compared to controls. The addition of GluA2-G-Gpep reverted this abnormal phenotype with longer, elongated mitochondria morphology. Scale Bar: 20 μm (left), 5 μm (right). **(b**) The average mitochondrial length was measured in each group. LPS-treated astrocytes had shorter mitochondrial length than non-treated astrocytes, while GluA2-G-Gpep treatment resulted in similar elongated mitochondria lengths as controls (n = 100 mitochondria from 3 different cultures per group, one-way ANOVA followed by Bonferroni *post hoc* test). **(c**) Cytokine assessments in primary astrocyte cultures under different treatments revealed that IL-1β, IL-6 and TNFα were significantly increased with LPS stimulation. No change was observed with IL-10, IL-17 and IFNγ levels. Moreover, GluA2-G-Gpep had no effects on regulating the release of these specific cytokines (n = 3 different cultures per group, one-way ANOVA followed by Bonferroni *post hoc* test). Cytokine experiments were performed in duplicates for each sample. Data are presented as mean ± SEM. **p < 0.01 vs. no treatment, ^++^*p* < 0.01 vs. LPS with GluA2-G-Gpep.

Mitochondrial functions within astrocytes are extremely crucial in maintaining proper astrocytic activity, especially with reactive astrocytes in response to CNS injuries^34^. For instance, astrocytic mitochondria has been shown to regulate calcium buffering activity of astrocytes, and provide ATP for various astrocytic functions^34^. Hence a dysfunction in spatial dynamics and positioning of mitochondria can greatly perturb all these behaviors. Motori *et al*. have demonstrated that there is an accelerated tendency of astrocytic mitochondrial fission, thus leading to their fragmentation, within highly proinflammatory brain areas^35^. To explore the changes in mitochondrial dynamics within neuroinflammation and the possible role of GluA2-GAPDH complex in its regulation, we analyzed mitochondria morphology and localization in primary astrocyte cultures with LPS stimulation and GluA2-G-Gpep treatment. The mitochondria within non-treated astrocytes displayed an elongated rod-like structure, while those in LPS-induced astrocytes formed a more circular shape. Addition of GluA2-G-Gpep corrected this abnormal phenotype (Fig. 7a). Correspondingly, mitochondrial length in reactive astrocytes was significantly shorter when compared to control and GluA2-G-Gpep treatment groups (No treatment: 72.99 ± 2.05; LPS: 47.25 ± 1.19; LPS + GluA2-G-Gpep: 67.25 ± 1.89 arb. units) (Fig. 7b). In terms of intracellular localization, we observed a higher number of mitochondria near the nuclear membrane within stimulated astrocytes, but they were more dispersed in control and peptide-treated cells (Fig. 7a). Our results indicate the presence of distinctive mitochondria morphology changes in reactive astrocytes, possibly via increased mitochondrial fission, and that the GluA2-GAPDH interaction may be involved in regulating mitochondrial dynamics.

### IL-1β, IL-6 and TNFα are significantly increased in reactive astrocytes induced by LPS, but GluA2-GAPDH disruption has no effect on cytokine release

An important signaling mechanism of astrocytes in response to injury is the production of many different cytokines and inflammatory mediators. Both pro- and anti-inflammatory cytokines have been reported to be secreted by astrocytes during neuroinflammation and MS^3,11,36^. Here, we measured various cytokine concentrations in the primary astrocyte culture media under LPS and GluA2-G-Gpep treatment. IL-1β, IL-6 and TNFα concentrations were significantly increased with LPS treatment, specifically IL-6 and TNFα displaying at least 1000-fold increase when compared to no treatment controls (Fig. 7c). However, IL-10, IL-17 and IFNγ were all nearly negligible, detected in the range of 0–2 pg/ml concentration in the presence of LPS (Fig. 7c). Surprisingly, GluA2-G-Gpep treatment had no significant effects on the concentrations of all cytokines analyzed in this study (Fig. 7c). We conclude that specific pro-inflammatory cytokines including IL-1β, IL-6 and TNFα were pronouncedly secreted in reactive astrocytes, but the GluA2-GAPDH complex is unlikely involved in the regulation of cytokine release.

### GluA2-GAPDH treatment reduces the elevated nuclear GAPDH, p53 and p53(S15) in LPS-stimulated astrocytes

**Figure 8:**
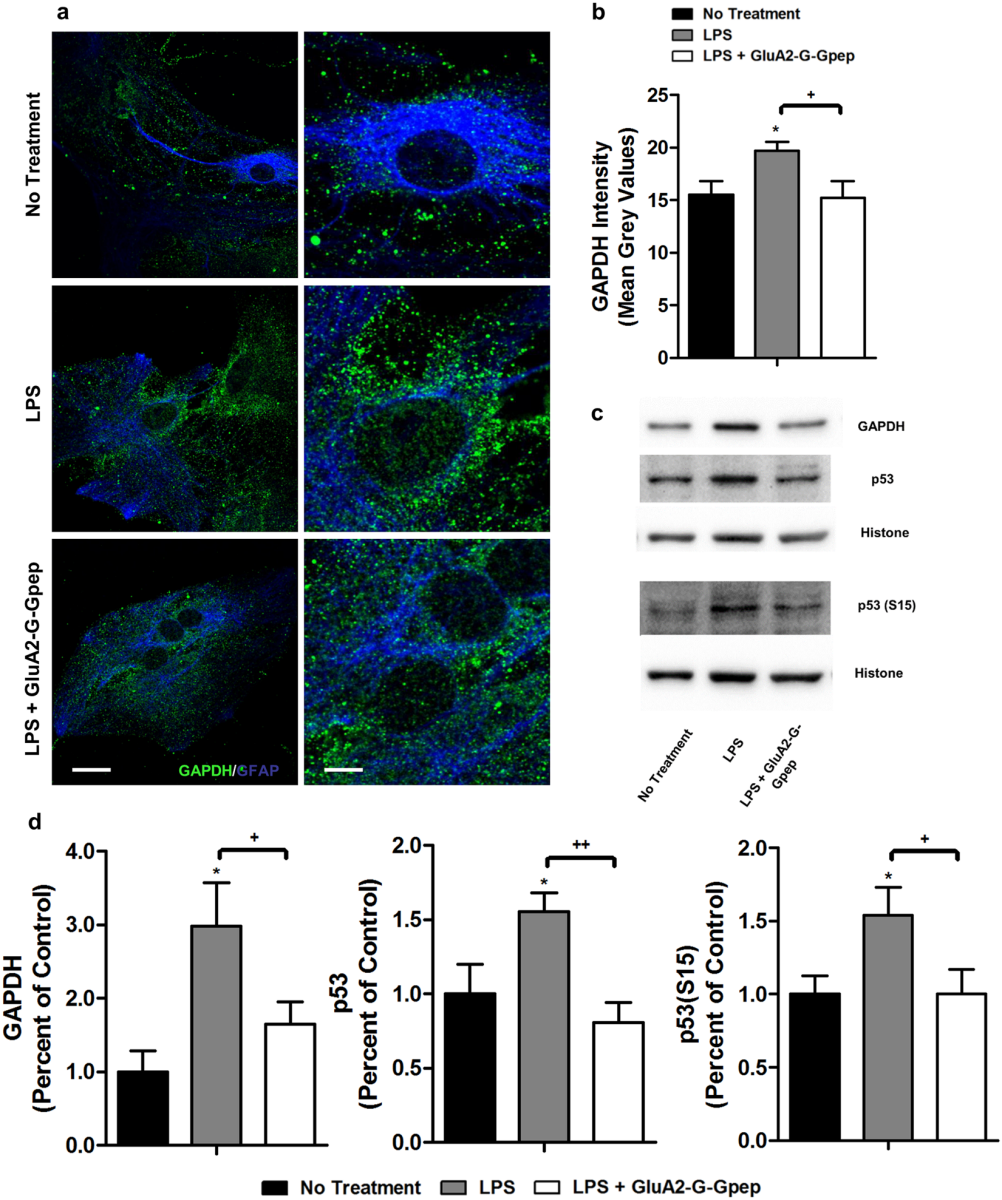
GluA2-G-Gpep treatment reduces the elevated nuclear GAPDH, p53 and p53(S15) in LPS-stimulated astrocytes. (**a)** Immunofluorescent images labeling GAPDH in primary astrocytes with different treatments. Higher magnification images are shown on the right. Astrocytes with LPS stimulation had significantly more GAPDH near nuclear membrane and within nucleus, but disruption of GluA2-GAPDH interaction with GluA2-G-Gpep prevented this effect. Scale Bar: 20 μm (left), 5 μm (right). **(b)** Quantification of GAPDH fluorescence intensity around the nucleus resulted in similar outcomes of higher expression in LPS-treated cells and decreased with GluA2-G-Gpep (n = 8 cells from 3 different cultures, one-way ANOVA followed by Bonferroni *post hoc* test). **(c,d**) Western blot analysis with nuclear fraction astrocyte proteins resulted in an increase of GAPDH protein levels, as well as p53 and p53 phosphorylated S15 with LPS. GluA2-G-Gpep treatment significantly reversed the expression to control levels (n = 3 different cultures per group, one-way ANOVA followed by Bonferroni *post hoc* test). Full-length Western blots are presented in Supplementary Fig. 5b. Data are presented as mean ± SEM. *p < 0.05 vs. no treatment, ^+^*p* < 0.05, ^++^*p* < 0.01 vs. LPS with GluA2-G-Gpep.

Our earlier studies have illustrated that the enhanced GluA2-GAPDH interaction promotes its internalization, leading to excess GAPDH in the nucleus^19^. Nuclear GAPDH can interact with p53, resulting in GAPDH-mediated upregulation of p53 expression and phosphorylation, and hence its transcriptional activity^37^. To examine whether this mechanistic pathway is associated with astrocytes, we examined nuclear fraction protein expression levels in primary astrocyte cultures. Immunocytochemistry revealed significantly more GAPDH located near the nuclear membrane and within the nucleus in LPS-induced astrocytes, but the addition of GluA2-G-Gpep partially reversed this effect (Fig. 8a). Similar results were confirmed by measuring the GAPDH fluorescence intensity in an area which is at a fixed distance around the nucleus (Fig. 8b). Consistently, astrocytic nuclear fractions of GAPDH, p53 and p53 phosphorylated S15 were all increased with LPS induction, and normalized back to control levels after peptide treatment (Fig. 8c,d). GAPDH within the cytoplasmic fraction of astrocyte proteins was also enhanced in LPS-reactive astrocytes, and GluA2-G-Gpep was able to decrease this effect. However, p53 levels remained similar for all groups (Supplementary Fig. 4a). Finally, we did not detect any GAPDH nor p53 in the membrane fraction of astrocytic proteins (Supplementary Fig. 4b). These findings present important clues to understanding the possible pathways involved in astrocytic changes regulated by GAPDH.

## Discussion

Neuroinflammation is a hallmark feature of CNS pathologies including MS. Increasing evidence is suggesting that microglia, peripheral immune cells and astrocytes are important mediators in this process. In particular, astrocytes are involved in a broad spectrum of different CNS functions, and research studies on astrocytic functions in MS have reported both deleterious and protective roles in disease progression^3,9,10^. In this study, we first established evidence to validate astrogliosis in the EAE mouse model and that treatment with GluA2-G-Gpep significantly reduced this state. This provides the basis that disruption of the GluA2-GAPDH interaction can affect astrocytic functions, which may partly explain the rescue of neurological symptoms with improved clinical scores of motor function as reported earlier using this peptide^21^. In addition, many astrocytic-specific proteins and genes expression have been reported to be associated with neuroinflammation and MS. Zamanian *et al*. conducted an extensive analysis on the changes in genes and associated pathways of reactive astrocytes, where they observed alterations in numerous genes and signaling pathways^8^. These findings consolidate the complex actions of astrocytes within MS.

In this study, we focused on the effects of GluA2-GAPDH disruption as a model to elucidate the distinct astrocytic changes regulated by this complex, which may also represent possible cellular mechanisms in ameliorating MS symptoms. Remarkably, GluA2-G-Gpep treatment reversed LPS-induced primary reactive astrocytes back to resting state. Morphologically, we noticed that F-actin organization pattern was disoriented in reactive astrocytes, possibly explaining their hallmark morphological changes. Berretta *et al*. found extensive conformational changes and F-actin rearrangement in mechanically-stretched reactive astrocytes^32^. Another group reported that F-actin in astrocytes treated with Fasudil (capable of reducing inflammation and demyelination in the EAE mice) exhibited a more globular shape^38^, corroborating to our findings with peptide treatment. Disrupting the GluA2-GAPDH interaction likely affects actin reorganization, which appears to play an important role in determining astrocyte morphological phenotypes.

In terms of astrocytic functions associated with MS, extensive evidence has characterized that compromised BBB integrity and glutamate excitotoxicity are two major components in MS pathogenesis. Consistent with our data, increased expression of AQP4 protein and mRNA was reported in LPS-induced reactive astrocytes and EAE mice^28^. Ikeshima-Kataoka *et al*. also described that AQP4 is dispersed in the cytoplasm of reactive astrocytes, similar to the observations in this study^28,39^. However, it is premature to conclude that disrupting GluA2-GAPDH can directly affect AQP4 localization based on our data, and further experiments are needed to address this issue. AQP4 activity and intracellular localization is regulated by phosphorylation from calcium-dependent kinases such as PKA, PKC and CaMKII^40^. As GluA2 subunits are the main determinants of AMPA receptor-mediated calcium permeability, alterations in calcium concentration would certainly affect activity of these kinases. Indirect effects of astrocytic changes to structural components such as integrins or annexins, or release of signaling factors including inflammatory cytokines can affect BBB integrity as well^41^.

Zeis *et al*. reported a transcriptional downregulation of *Eaat1* in LPS-treated astrocytes, but an upregulation of *Eaat2*^42^. Interestingly, they also found *Eaat2* was downregulated in chronic MS normal appearing cortical gray matter, in which the authors explained that this discrepancy could be due to the lack of their normal cellular counterparts such as neurons and oligodendrocytes^42^. Furthermore, Mandolesi and colleagues described a downregulation of EAAT1 in EAE cerebellum^43^, while an enhanced EAAT1 immunoreactivity and glutamate transport capacity of reactive astrocytes was observed in other studies^44^. As suggested by Schreiner, these contradictory results may be due to differences in the degree of astrogliosis. In our study, LPS treatment was challenged at a concentration of 1 μg/ml for 48 hours, compared to Zeis *et al*. which used 10 ng/ml for 24 hours. Moreover, there could be regional differences in astrocyte functions of glutamate uptake that respond to specific local signals. This may also explain why glutamate uptake functions did not respond in a similar manner despite detecting significant EAAT1 and EAAT2 expression reduction in inflammatory conditions with peptide treatment. Transporter activity is regulated at multiple levels, including protein expression, cell surface trafficking, protein binding, and by phosphorylation and other direct modifications^45^. A detail examination on how GluA2-GAPDH can cause the changes in AQP4, EAAT1, EAAT2 expression and their related functions of BBB permeability and glutamate uptake associated with MS would be promising for future research.

Recent transcriptome analysis of reactive astrocytes and mass-spectrometry proteomic data revealed the upregulation of crucial genes and proteins in the metabolic pathways, implying the importance of energy metabolism in neuroinflammation^8^. An abundance of mitochondria exists in astrocytes to supply energy for their broad physiological functions, and mitochondrial dysfunction has been linked to neurodegenerative diseases^35,46^. Motori *et al*. showed that astrocytic mitochondria within inflammatory brain area had an accelerated mitochondrial fission, leading to their fragmentation. Dynamin-related protein 1 (Drp1) is a primary regulator of mitochondrial fission, where it binds to p53 to induce mitochondria-associated cell death^47^. We previously demonstrated that the internalized GluA2-GAPDH complex can lead to excess GAPDH binding to p53, resulting in the upregulation of p53 expression and phosphorylation^37^. Indeed, nuclear fractions of astrocyte GAPDH, p53 and p53(S15) were all significantly increased with LPS induction, but perturbing the GluA2-GAPDH interaction lowered their expression levels. The increase in p53 expression and phosphorylation can activate PTEN, a negative regulator of AKT, which subsequently lead to less inactivation/phosphorylation of Foxo3a (substrate of AKT)^48,49^. Since Foxo3a transcriptionally upregulates AQP4^50^, this may represent a possible pathway in which how GluA2-GAPDH disruption can produce alterations in AQP4 expression. Future investigations on the role of GAPDH and p53 pathways would be needed to fully understand the exact mechanisms.

Finally, the dual deleterious and protective roles of astrocytes in MS can be strongly attributed to the changes of cytokines as they secrete both pro- and anti-inflammatory cytokines. Choi *et al*. conducted a comprehensive secretome profile of cytokines in stimulated human astrocytes, and found that IL-1β, IL-6 and TNFα were all elevated while no alterations with IL-10, IL-17 and IFNγ, completely agreeing with our cytokine profile^51^. We did not detect any cytokine changes with GluA2-G-Gpep treatment, suggesting that the GluA2-GAPDH complex is not involved in regulating cytokine release in astrocytes. In our earlier study, we found that the pro-inflammatory IL-17 and IFNγ levels were reduced in supernatants of EAE mice treated with GluA2-G-Gpep^21^. We speculate that other glial cells such as microglia or perhaps an indirect secondary effect resulting from other pathways are responsible for this reduction. Nevertheless, our results provide knowledge on the specific cytokines released from astrocytes during neuroinflammation and that the GluA2-GAPDH interaction may not be directly involved in governing this process.

In recent years, astrocytes have gained much attention for their roles in neuroinflammatory diseases including MS. Our findings support the involvement of the GluA2-GAPDH complex in regulating different astrocytic functions and that these effects are mediated by GluA2 and GAPDH cellular pathways instead of affecting AMPA receptor functions. Future experiments are required to decipher the exact pathways for each function and examine the effects on other cells including neurons, microglia and oligodendrocytes in relation to astrocytes. The broad spectrum of astrocytic functions, especially the capability of playing both protective and neurotoxic roles is intriguing. Recent work by Liddelow *et al*. has shown that reactive astrocytes exist in two different subtypes, namely A1 and A2 astrocytes^52^. Given our results in presenting novel information on the distinct astrocyte changes regulated by the GluA2-GAPDH complex associated with MS, it would be interesting to investigate their association with A1 and A2 astrocytes in the future.

## Methods

### Mice

For EAE experiments, female C57/BL6 mice (8–12 weeks of age) were purchased from Charles River Laboratories. For all other cellular and biochemistry experiments, mice were bred at the Centre for Addiction and Mental Health (CAMH) (Toronto, Canada). All mouse protocols were approved by the CAMH Animal Care Committee and methods were carried out in accordance with the approved guidelines.

### GluA2-G-Gpep peptide synthesis

GluA2-G-Gpep (YK-41) peptide was synthesized by Biomatik Corporation (Cambridge, Canada). The cell membrane transduction domain of HIV-1 TAT protein sequence (YGRKKRRQRRR) was fused to the N-terminus of the peptide, facilitating its intracellular delivery. The final protein sequence of the GluA2-G-Gpep used in this study was YGRKKRRQRRR-YYQWDKFAYLYDSDRGLSTLQQVLDSAAEK. The peptide was further purified by high-performance liquid chromatography to 98% purity, dissolved in 0.9% saline and aliquots were stored at −80 °C.

### Induction of EAE and peptide treatment

EAE was induced in mice (8–12 weeks of age) as previously described^21^. Briefly, mice were injected subcutaneously with 200 µL of recombinant MOG_35–55_ (Biomatik Corporation), emulsified in incomplete Freund’s adjuvant (Sigma, Oakville, Canada) and supplemented with 4 mg/mL Mycobacterium tuberculosis (strain H37Ra, BD Biosciences, Mississauga, Canada) at four different sites with 50 µL per site. 200 ng of pertussis toxin (List Biological Laboratories, Inc., Campbell, CA, USA) was injected intraperitoneally (i.p.) on day 0 and 2 after immunization. Mice were assessed for clinical signs of disease at the beginning of immunization. 3 nmol/g of GluA2-G-Gpep or TAT-control peptide was subsequently injected (i.p.) daily starting from day 10 of immunization for 18 days until day 28. Our group had previously shown that this peptide concentration effectively disrupted the GluA2-GAPDH interaction^21^.

### Primary astrocyte culture preparation and treatment

Cortical tissues from postnatal day 1–3 (P1-3) mouse brains were dissected out, incubated with 0.25% trypsin for 15 min at 37 °C, and dissociated by mechanical trituration. Cells were plated on T25 cell culture flasks, and grown in DMEM with 10% fetal bovine serum (FBS), 100 U/ml penicillin and 100 μg/ml streptomycin in an incubator (37 °C, 5% CO_2_) until astrocyte monolayers were confluent. Half of the DMEM was replaced every 3–4 days. To remove microglia and oligodendrocytes from mixed glial cultures, flasks were shaken for 24 hours at 200 rpm on an orbital shaker. Afterwards, versene solution and 2.5% trypsin were added to completely disaggregate astrocytes. Purified astrocytes were resuspended in DMEM/10% FBS and plated on either glass coverslips or 60 mm culture dishes, and grown until approximately 50% confluency for immunocytochemistry or completely confluent for biochemistry experiments. The purity of astrocytes was confirmed with GFAP/DAPI labeling. Astrocyte cultures were challenged with 1 μg/ml of lipopolysaccharide **(**LPS) from *Escherichia coli* 0111:B4 (Sigma, L2630) dissolved in PBS for 48 hours to induce activation. 10 μM of GluA2-G-Gpep or TAT-control peptide was added to LPS-treated astrocyte cultures for 3 hours before processing for immunocytochemistry, Western blot experiments, glutamate measurements or cytokine assessments.

### Immunohistochemistry

Lumbosacral spinal cords from EAE mice of different groups were dissected out, fixed in 4% paraformaldehyde (PFA) overnight at 4 °C, cryoprotected in 30% sucrose and frozen at −80 °C before further processing. 20 μm-thickness frozen coronal sections were cut using a microtome cryostat system (Bright Instruments 5030). Free floating sections were initially blocked in 5% fetal bovine serum, 1% Triton X-100, 0.5% Tween 20 and 1% skim milk in 0.1 M PBS for 2 hours at room temperature to reduce non-specific binding. This was followed by incubation with primary antibodies of anti-GFAP (1:200, Dako Z0334, Glostrup, Denmark), anti-AQP4 (1:200, Abcam, ab9512, Cambridge, MA, USA), anti-mouse IgG (1:50, Sigma, A9044), anti-Occludin (1:100, Santa Cruz Biotechnology, sc-5562, Dallas, TX, USA), anti-EAAT1 (1:200, Abcam, ab416), anti-EAAT2 (1:200, Abcam, ab41621), anti-GluA2 (1:200, Novus Biologicals, NBP1-46490, Oakville, Canada) and anti-GAPDH (1:200, Millipore Canada, MAB374, Etobicoke, Canada) overnight at 4 °C. Alexa 488- or 594-conjugated secondary antibodies (1:200; Thermo Fisher Scientific, Burlington, Canada) in blocking solution were added for 2 hours at room temperature. DAPI was used to stain nuclei.

### Immunocytochemistry

Cultured astrocytes were fixed in 4% PFA/4% sucrose, permeabilized with 0.1% Triton X-100 in 0.1 M PBS for 15 min, and blocked for 1 hour with 1% bovine serum albumin in PBS at room temperature. Similarly, they were incubated with primary antibodies overnight at 4 °C and secondary antibodies for 2 hours at room temperature. The primary antibodies used include anti-GluA2 (1:500, Novus Biologicals, NBP1-46490), anti-GAPDH (1:200, Millipore Canada, MAB374), anti-GFAP (1:500, Dako Denmark, Z0334), anti-EAAT1 (1:200, Abcam, ab416), anti-EAAT2 (1:200, Abcam, ab41621) and anti-AQP4 (1:200, Abcam, ab9512). Alexa 488- or 594-conjugated secondary antibodies were used for detection of primary antibodies. Alexa 488 phalloidin (Thermo Fisher Scientific, A12379), mitotracker red (Thermo Fisher Scientific, M7512) and DAPI were also applied to stain F-actin, mitochondria and nuclei respectively.

### Time-lapse video imaging

Astrocyte cultures were plated on 35 mm glass bottom dishes from MatTek Corporation (Ashland, MA, USA) and grown for 2 days. 1 μg/ml of LPS was added into the media for 48 hours followed by 10 μM of GluA2-G-Gpep. Differential interference contrast (DIC) images were captured using the Vivaview FL incubator microscope (Olympus, Toronto, Canada). The recording parameters for image capturing were set at 10-minute intervals for 60 hours for analysis of astrocyte morphology.

### Immunohistochemistry analysis

Fluorescent images were captured at 10 × magnification using the Zeiss LSM510 Meta confocal microscope, converted to grey-scale and normalized to background staining. A two-dimensional random sampling window approach on regions of interest (ROI) was employed to provide accurate estimates of cell densities, fluorescent intensity and occupancy values. Fluorescent cells within each ROI were counted using the ITCN plugin for ImageJ (https://imagej.nih.gov/ij/). A fixed parameters of cell width and threshold is set where only cells that reach the minimum signal will be counted. As for fluorescent occupancies, images were converted to a pre-calibrated black and white threshold scale using ImageJ, in which fluorescent intensities that reach a standard threshold become black while the rest remain white. Therefore, quantification of GFAP^+^ astrocytes reactivity was measured as the percentage of area occupied by fluorescent-labeling in each ROI. Mean grey values were also used to define fluorescence signal intensity with images converted to a pre-set grey scale (ImageJ). All image-capturing and threshold parameters were kept the same for each measurement between comparing groups. ROIs of fixed area (200 × 250 um^2^) were positioned over the dorsal, intermediate and ventral regions of spinal cord grey matter for each analysis, while whole spinal cord sections were outlined for intensity analyses. Fluorescent heat maps of EAAT1 and EAAT2 were shown to illustrate intensity and distribution.

### Immunocytochemistry analysis

All images were captured using a confocal microscope (Olympus FluoView FV1200) at either 10 × or 60 × magnification. For 10 × images, GFAP^+^ cells were manually counted determined by more than 50% fluorescence of the cell, and the percentage was calculated by dividing the total number of cells in the field of view. As for morphological analyses of individual astrocytes, the cell perimeter was first outlined. Mean grey values of GFAP intensity were measured similarly as previously described, and surface area was also quantified with ImageJ. The number of primary astrocyte filopodia, defined as distinct processes originate from the cell body was counted. In addition, astrocytic mitochondrial length was measured with ImageJ. Heat maps of AQP4 fluorescent intensity were used to delineate its intracellular localization. Finally, GAPDH fluorescence intensity was measured in selected regions of fixed distance around the nucleus.

### Co-immunoprecipitation and Western blot

Co-immunoprecipitation and Western blot procedures were performed according to previous protocols^53,54^. In brief, for co-immunoprecipitation, 500 µg of solubilized proteins extracted from primary astrocyte cultures in RIPA buffer (50 mM Tris-HCl pH 7.4, 150 mM NaCl, 2 mM EDTA, 1 mM PMSF with 1% Igepal CA-630, 1% sodium deoxycholate, 1% Triton X-100 and protease inhibitor cocktail (5 μl/100 mg of tissue; Sigma, P8340) were incubated in the presence of anti-GluA2 antibody (Novus Biologicals, NBP1-46490) or control IgG (1–2 µg) for 4 hours at 4 °C, followed by the addition of protein A/G plus agarose (Santa Cruz Biotechnology) overnight. Pellets were washed, boiled in sodium dodecyl sulfate (SDS) sample buffer for 5 min and subjected to SDS-polyacrylamide gel electrophoresis. Proteins were subsequently transferred onto nitrocellulose membranes and Western blotted with an anti-GAPDH antibody (Millipore, MAB374). Western blot analysis was performed using 30 µg of proteins from mouse spinal cord tissues or 50–100 µg of proteins from primary astrocyte cultures of all groups. Astrocyte nuclear proteins were extracted using the Membrane, Nuclear and Cytoplasmic Protein Extraction Kit according to manufacturer’s protocol (Bio Basic, BSP002, Markham, Canada). Blots were incubated with primary antibodies overnight at 4 °C, then with horseradish peroxidase-conjugated secondary antibodies for 2 hours at room temperature. The antibodies used were anti-GFAP (Dako Denmark, Z0334), anti-vimentin (Abcam, ab92547), anti-GAP43 (Abcam, ab16053), anti-14-3-3ε (Proteintech, 11648-2-AP, Rosemont, IL, USA), anti-NCAM1 (Proteintech, 14255-1-AP), anti-α-tubulin (Millipore, 05–829), anti-EAAT1 (Abcam, ab416), anti-EAAT2 (Abcam, ab41621), anti-AQP4 (Abcam, ab9512), anti-β-actin (Ambion, AM4302, Fisher Scientific), anti-p53 (Cell Signaling Technology, 2524, Davers, MA, USA), anti-p53(S15) (Abcam, ab1431), anti-histone H3 (Abcam, ab1791) and anti-Na^+^K^+^ ATPase (Abcam, ab76020). Protein bands were visualized with enhanced chemiluminescence reagents (Amersham Biosciences, Buckinghamshire, UK). The intensity of all resulting bands was quantified by densitometry using ImageJ. Western blot bands were normalized to loading bands, expressed as a percentage of sham or no treatment groups and conducted in duplicates for each sample.

### Glutamate measurement

Glutamate concentration in the media of primary astrocyte cultures under different treatments were assessed using the Glutamate Assay kit (Colorimetric) (Abcam). 50 μM of glutamate was added at the beginning and 50 μl of media was obtained every 5 min for glutamate measurement in a 15-minute time span. Glutamate concentration measured at each time point was expressed as a ratio of the concentration at time 0 for each batch of cells. Glutamate uptake percent was calculated by subtracting the difference in glutamate concentration between time 0 and 15 min, and dividing by the starting concentration.

### Cytokine assessments

Multiplexed microsphere suspension ELISA platform (Luminex, US) was used to assess the levels of expression of inflammatory interleukins (IL-1β, IL-6, IL-10, IL-17, IFNγ and TNFα) according to the manufacturer’s instructions (Bio-Plex, BioRad, Mississauga, Ontario, Canada). Blank controls, cytokine standards and samples were prepared in duplicates in a 96-well plate and incubated with beads, detection antibodies and streptavidin-PE. Fluorescent intensity of the beads was measured and the four-parameter logistic curve fitting was used for data analysis.

### Genome-wide association study

Summary data from two genome-wide association studies (GWAS) of MS were extracted from the dbGAP database. The dataset phs000171.pha002861 s was derived from logistic regression analyses of the Baranzini *et al*. GWAS data on 978 MS cases and 883 controls of European ancestry using the Illumina Sentrix Human-Hap550 BeadChip, including site (US, Amsterdam, Basel), sex, and DRB1*1501+/− as covariates^55^. The dataset phs000139.pha002854 s was derived from the GWAS analysis conducted by the International Multiple Sclerosis Genetics Consortium^56^ on 960 trios genotyped on the Affymetrix GeneChip Human Mapping 500 K Array. Candidate gene regions including 10 kilobases upstream and downstream were extracted from the summary files using R version 3.2.0.

### Statistical analysis

The Student’s two-tailed *t*-test was performed to determine statistical differences between sham control and EAE groups for immunohistochemistry and Western blot analyses. The one-way or two-way ANOVA (GraphPad Prism 5) followed by Bonferroni’s correction for multiple testing was used for multiple group comparisons in all other experiments. All images were blinded prior to analysis. Data were expressed as mean ± standard error of mean (SEM). A significance level of *p* < 0.05 was used for all analyses.

## Electronic supplementary material

Supplementary Figures
