# Supplementary material for: Specific Alterations in Astrocyte Properties via the GluA2-GAPDH Complex Associated with Multiple Sclerosis
Source: Sci Rep. 2018 Aug 27;8:12856. doi: 10.1038/s41598-018-31318-4 (PMC6110783; doi:10.1038/s41598-018-31318-4)
Supplement: Supplementary file 1 — Supplementary Figures [file 41598_2018_31318_MOESM1_ESM.pdf]

Specific Alterations in Astrocyte Properties via the GluA2-GAPDH Complex Associated with Multiple Sclerosis

[1]Frankie HF Lee, [1]Hailong Zhang, [1]Anlong Jiang [1,2,3,4]Clement C Zai, and [1,2,5]Fang Liu*

[1]Campbell Family Mental Health Research Institute, Centre for Addiction and Mental Health, Toronto, Ontario, Canada. M5T 1R8

[2]Department of Psychiatry, [3]Institute of Medical Science, [4]Laboratory Medicine and Pathobiology, [5]Physiology, University of Toronto, Toronto, Ontario, Canada. M5T 1R8

Supplementary Information

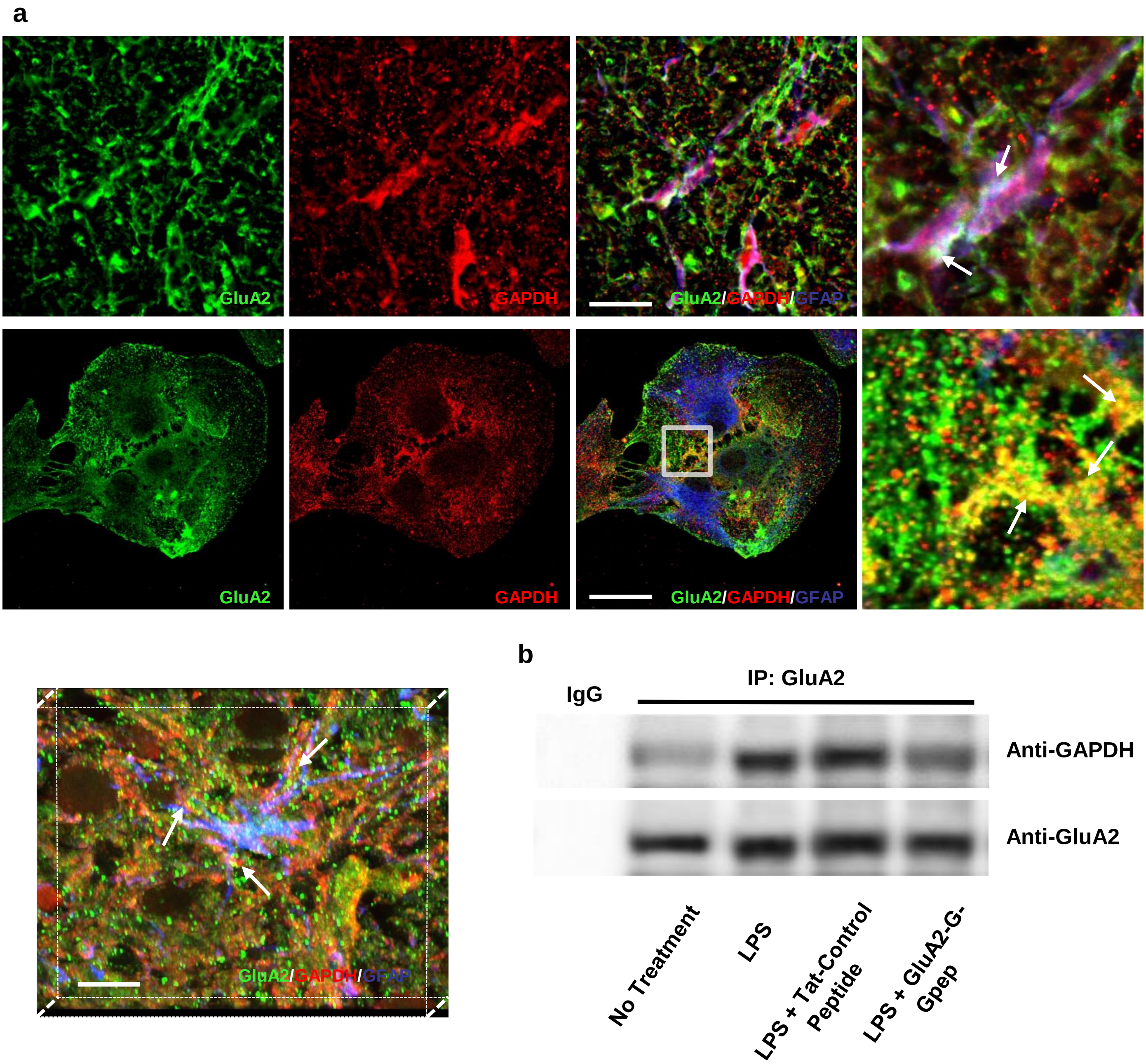

Supplementary Fig. 1. GluA2-GAPDH interaction is enhanced in LPS-induced reactive astrocytes, but GluA2-G-Gpep treatment effectively disrupted this complex formation. (a) Immunohistochemistry on mouse spinal cord sections (top) and immunocytochemistry with primary astrocyte cultures (bottom) revealed colocalization of GluA2 and GAPDH in GFAP-positive astrocytes. Scale bar: 50µm (top), 20µm (bottom). Higher magnification of images are shown in the right panel. A combined z-stack image of mouse spinal section is also represented below. White arrows indicate colocalization of GluA2 and GAPDH in GFAP-astrocytes. **(b)** Co-immunoprecipitation of primary astrocytes showed a prominent increase in GluA2-GAPDH interaction with LPS stimulation when compared to non-treatment groups, while GluA2-G-Gpep treatment significantly disrupted this interaction. Experiments were performed in duplicates with three different cultures.

Supplementary Figure 1

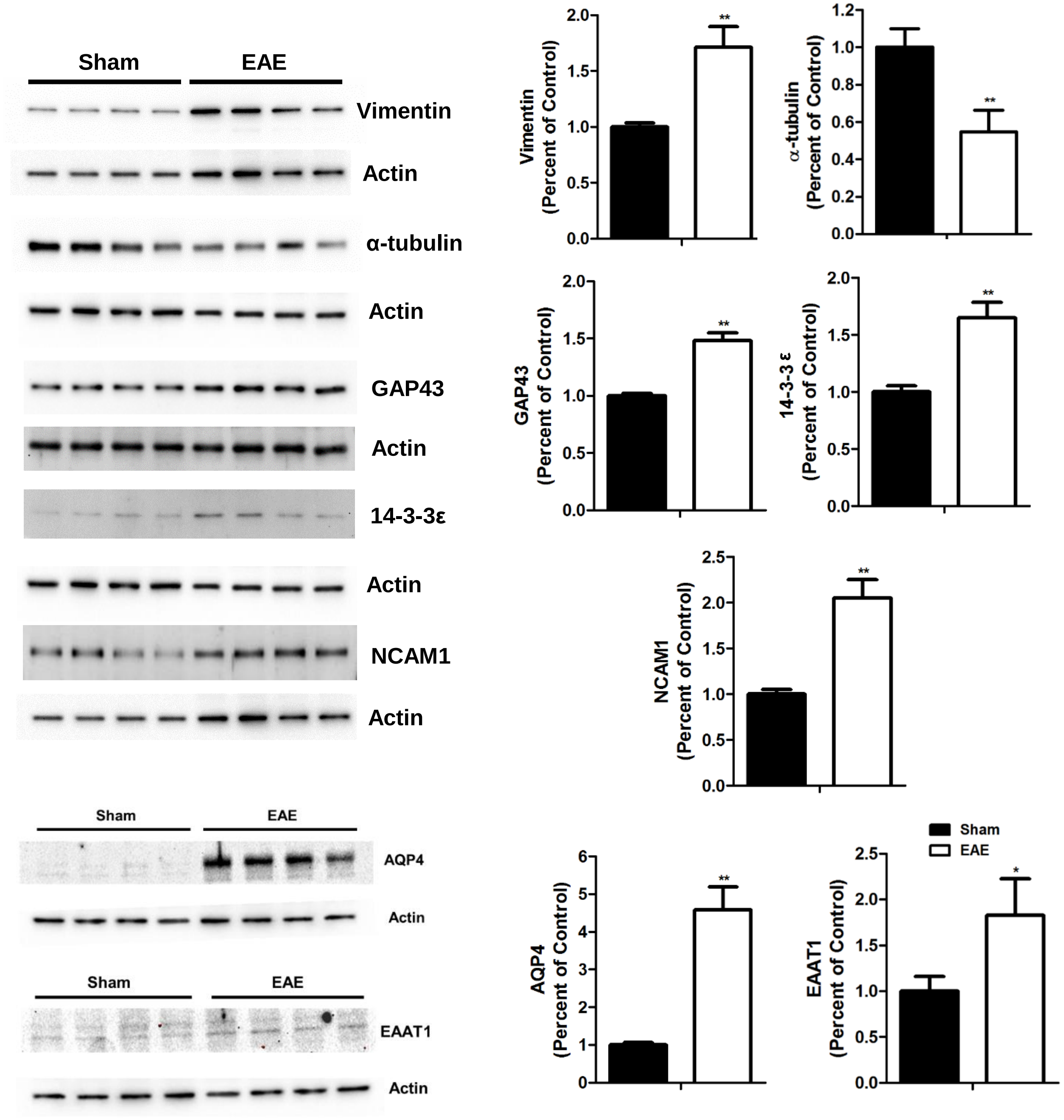

Supplementary Fig. 2. Different proteins show significant expression changes in the EAE mice. Western blot experiments were performed to quantify various proteins that have been shown to be associate with astrocytic functions in the EAE mouse model. These proteins include vimentin, α-tubulin, GAP43, 14-3-3ε, NCAM1, AQP4 and EAAT1. We found that all proteins except α-tubulin were significantly increased in the EAE group when compared to sham (n=4 spinal cords per group, two-tailed t-test). Full-length blots are shown and quantification of protein expression was normalized with actin loading controls and expressed as a percentage of sham groups. The same blots are represented with loading controls. Data are presented as mean ± SEM. *$p<0.05$, **$p<0.01$.

Supplementary Figure 2

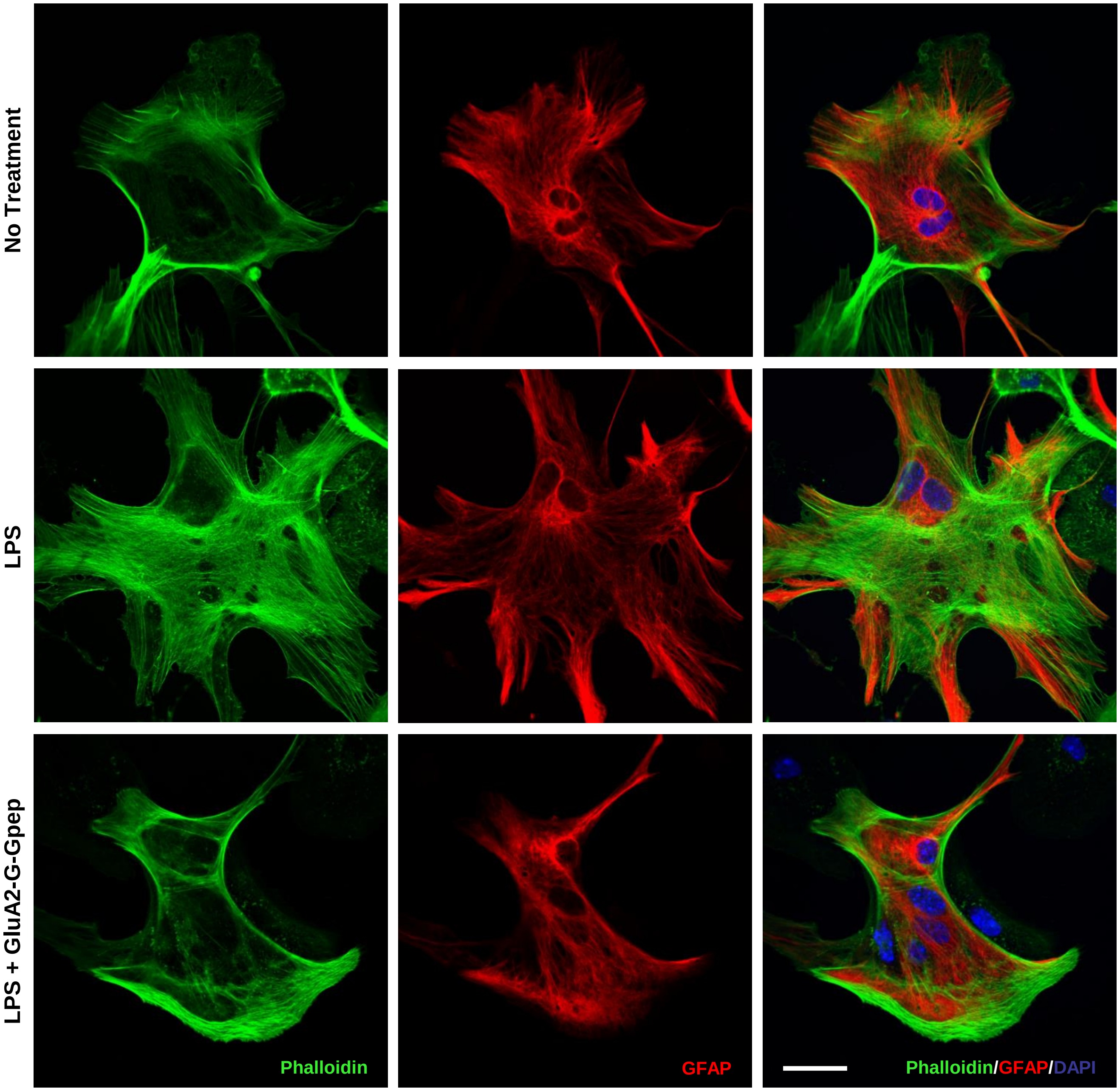

Supplementary Fig. 3. LPS-challenged reactive astrocytes show distinct actin organization pattern, while disrupting GluA2-GAPDH interaction reorganizes actin fibres. Fluorescent images showing F-actin fibres (labeled with phalloidin) in primary astrocyte cultures under various treatment. Normal astrocytes displayed a distinct well-organized ring structure at the outer edge of the cell, but F-actin in LPS-induced reactive astrocytes were more localized in the middle cytoplasmic region. Surprisingly, actin is reorganized in astrocytes with GluA2-G-Gpep treatment back to a normal ring shape pattern. Scale Bar: 20µm.

Supplementary Figure 3

a

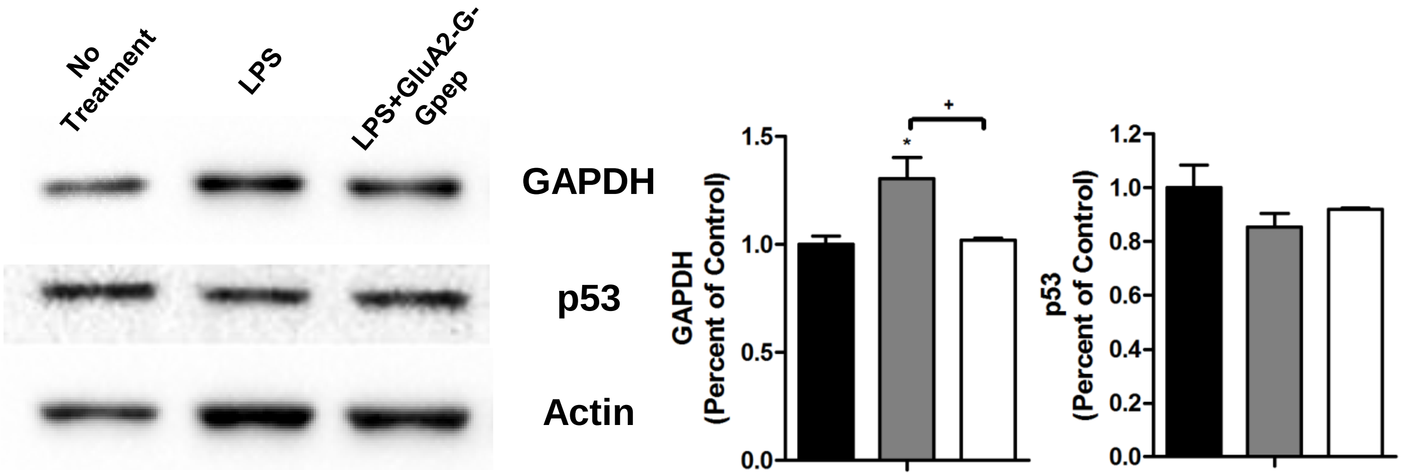

b

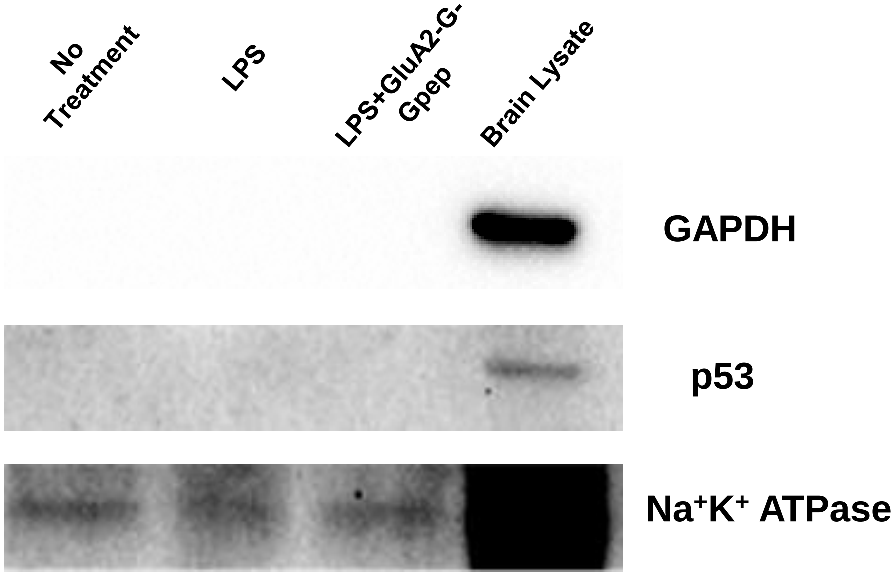

Supplementary Fig. 4. GluA2-G-Gpep treatment significantly reduces the enhanced GAPDH in the cytoplasmic protein fraction of LPS-induced astrocytes, but no change is observed with p53. (a) Western blot results of astrocyte cytoplasmic proteins showed a significant increase in GAPDH in astrocytes with LPS stimulation, but GluA2-G-Gpep reduced GAPDH level back to normal. There was no change in p53 expression levels for all groups (n=3 different cultures per group, one-way ANOVA followed by Bonferroni *post hoc* test). Quantification of protein expression was normalized with actin loading controls and expressed as a percentage of no treatment groups. Data are presented as mean ± SEM. $*p<0.05$ vs. no treatment, $^{+}p<0.05$ vs. LPS with GluA2-G-Gpep. **(b)** Membrane proteins of astrocytes for different groups did not show any presence of GAPDH or p53. Full-length blots are presented in Supplementary Fig. 5c and d. $Na^{+}K^{+}$ ATPase was used as a loading control, while a lane of brain lysate acted as a positive control. The same blots are represented with loading controls.

Supplementary Figure 4

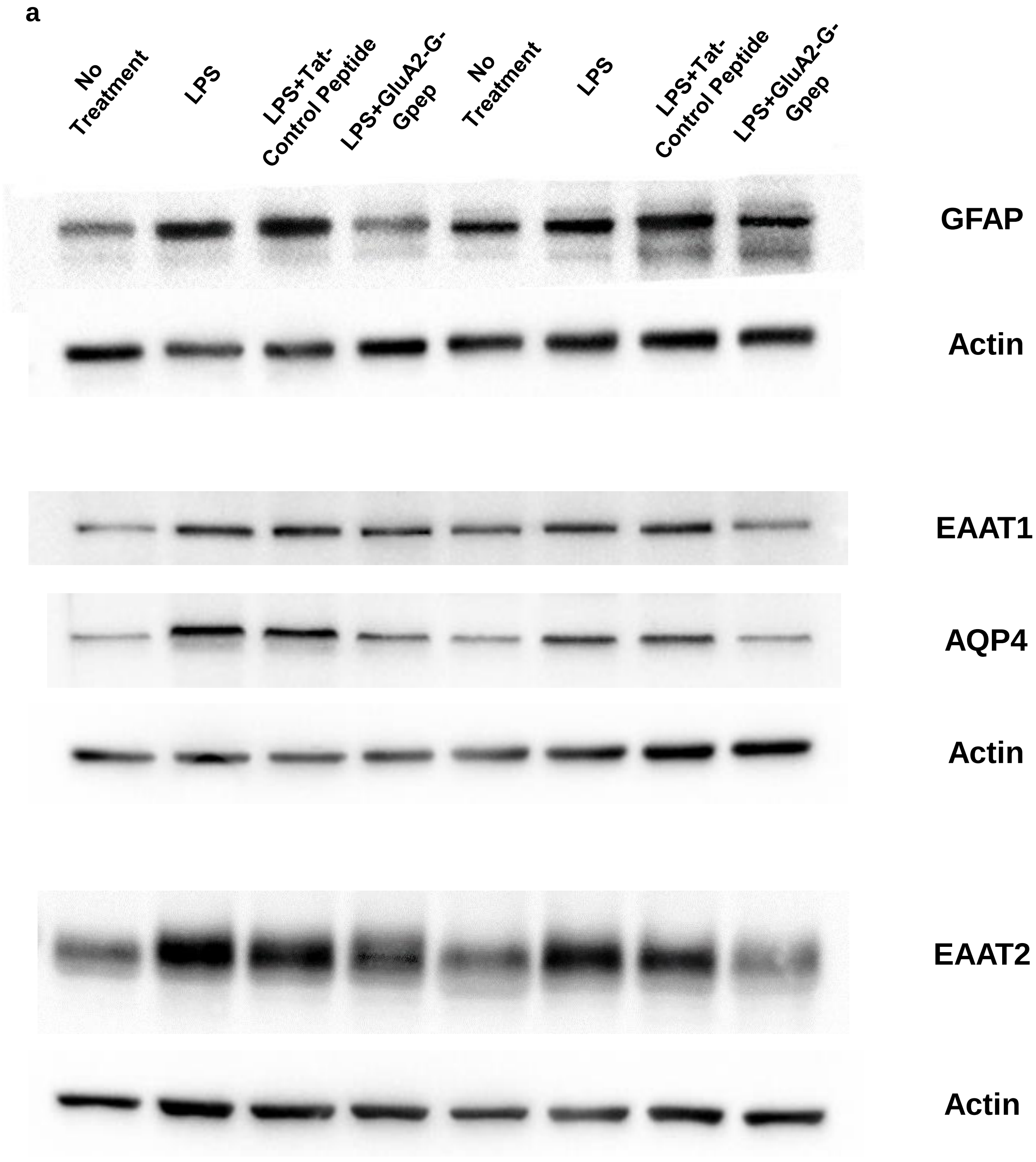

Supplementary Figure 5

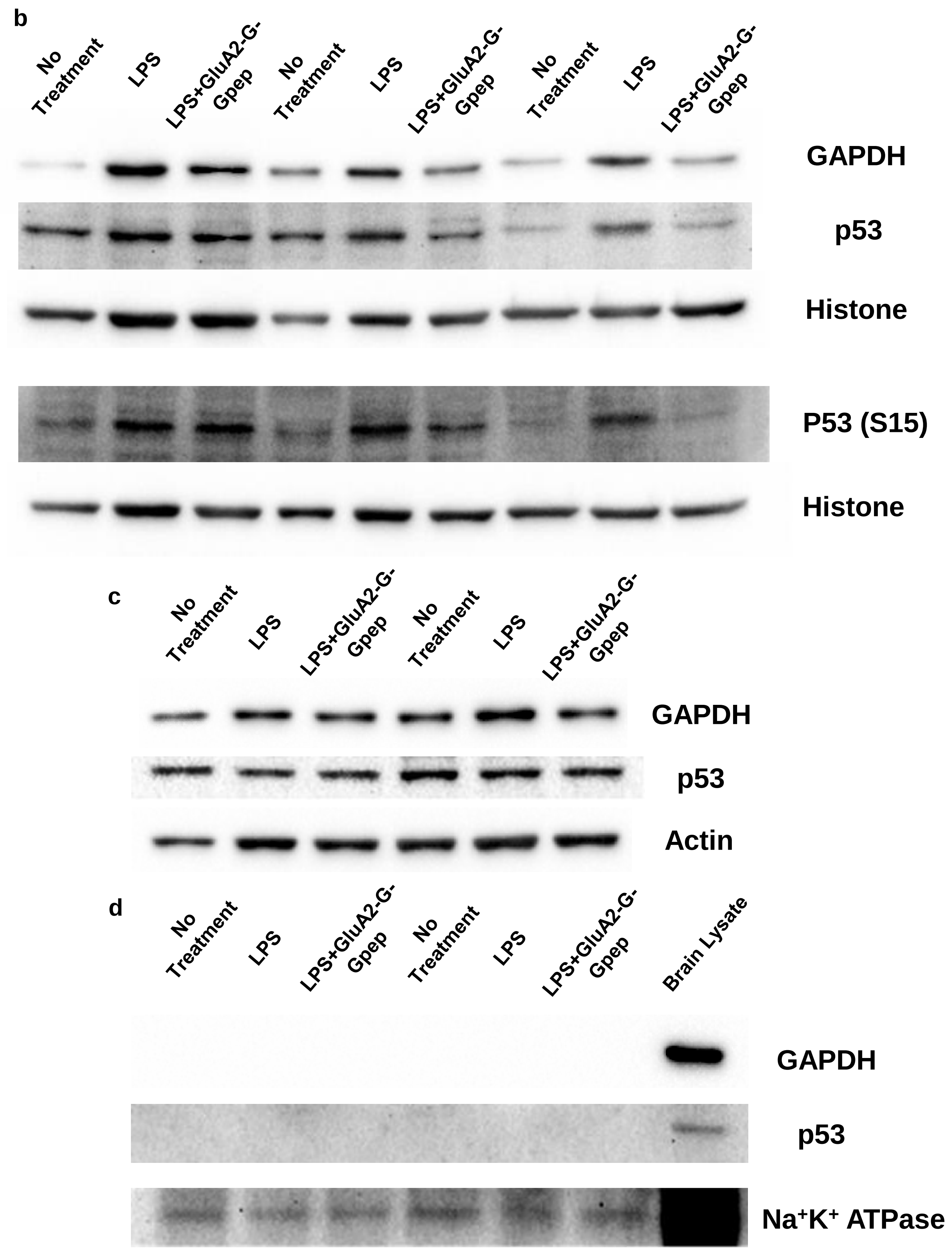

Supplementary Figure 5 (cont.)

Supplementary Fig. 5. Full-length Western blots. (a) Representative full-length Western blots from Fig. 4e, 5b, 6b and 6d, showing GFAP, AQP4, EAAT1 and EAAT2 expression using proteins from astrocyte cultures with no treatment, LPS, LPS with Tat-control peptide, and LPS with GluA2-G-Gpep treatment. **(b)** Similarly, images of full-length Western blots from Fig. 8c showing GAPDH, p53 and p53 (S15) protein expression in astrocyte nuclear proteins under the specified treatment. Cytoplasmic **(c)** and membrane **(d)** proteins of astrocytes of GAPDH and p53 are shown. Actin was used as a loading control for **(a)** and **(c),** histone for (**b**), and $Na^{+}K^{+}$ ATPase for **(d)**. A lane of brain lysate was also used as a positive control for membrane proteins. The same blots are represented with loading controls.

Table 1. Selected genes with astrocyte properties showing consistent association of SNPs in GWAS

Findings from two independent GWAS showing nominal association of markers in our genes of interest with MS				
SNP ID	***P.value***	***Odds Ratio***	***Gene***	***Dataset***
rs1438367	0.03523	1.229947	GAP43	International Multiple Sclerosis Genetics Consortium *et al.*, 2007
rs7780081	0.0248	1.186709	AQP1	
rs1715952	0.02958	1.268456		
rs283367	0.0053	1.152464	GAP43	Baranzini *et al.*, 2009

Supplementary Table 1
